# Comparative Physiological, Biochemical, and Leaf Proteome Responses of Contrasting Wheat Varieties to Drought Stress

**DOI:** 10.3390/plants13192797

**Published:** 2024-10-05

**Authors:** Sellwane J. Moloi, Ali O. Alqarni, Adrian P. Brown, Tatenda Goche, Nemera G. Shargie, Makoena J. Moloi, Arun Gokul, Stephen Chivasa, Rudo Ngara

**Affiliations:** 1 Department of Plant Sciences, University of the Free State, Qwaqwa Campus, P. Bag X13, Phuthaditjhaba 9866, South Africa; moloisj@ufs.ac.za (S.J.M.); gokula@ufs.ac.za (A.G.); 2 Department of Biosciences, Durham University, South Road, Durham DH1 3LE, UK; ali.alqarni@durham.ac.uk (A.O.A.); a.p.brown@durham.ac.uk (A.P.B.); tatenda.goche@durham.ac.uk (T.G.); stephen.chivasa@durham.ac.uk (S.C.); 3 Department of Crop Science, Bindura University of Science Education, P. Bag 1020, Bindura, Zimbabwe; 4 Agricultural Research Council-Grain Crops, P. Bag X1251, Potchefstroom 2520, South Africa; shargien@arc.agric.za; 5 Department of Plant Sciences-Botany Division, University of the Free State, 205 Nelson Mandela Drive, Bloemfontein 9301, South Africa; moloimj@ufs.ac.za

**Keywords:** *Triticum aestivum*, drought, proline, oxidative stress, lipid peroxidation, antioxidant enzymes, proteomics, iTRAQ, photosynthesis, gene expression

## Abstract

Drought stress severely affects crop productivity and threatens food security. As current trends of global warming are predicted to exacerbate droughts, developing drought-resilient crops becomes urgent. Here, we used the drought-tolerant (BW35695) and drought-sensitive (BW4074) wheat varieties to investigate the physiological, biochemical, and leaf proteome responses underpinning drought tolerance. In response to drought, the tolerant variety had higher osmolyte accumulation and maintained higher leaf water content than the sensitive variety. BW35695 also had an enhanced antioxidant enzyme capacity and reduced reactive oxygen species (ROS), resulting in diminished membrane lipid damage, as reflected by malondialdehyde content. Proteomic analysis revealed that drought-induced differential expression of proteins involved in diverse biological processes in both wheat varieties, including primary and secondary metabolism, protein synthesis/folding/degradation, defense/ROS detoxification, energy, transcription, and cell structure. Notably, photosynthesis emerged as the most enriched biochemical process targeted for suppression in the drought-tolerant BW35695 wheat, but not in drought-sensitive BW4074, possibly as a survival strategy for averting cell damage inflicted by photosynthesis-derived ROS. Additionally, protein synthesis-related proteins were highly upregulated in BW35695, presumably to drive cell-wide stress-adaptive responses. The protein network identified here will be useful in further studies to understand the molecular basis for divergent drought response phenotypes in crops.

## 1. Introduction

Drought stress threatens global crop production, exposing vulnerable populations to food insecurity [1]. In Sub-Saharan Africa, global warming is projected to intensify the prevalence of hot and dry spells, which are currently causing devastating yield losses, across the region [2,3]. Consequently, more drought-tolerant crops are required to meet the global food demand under the changing climate [4]. This has led to growing research interest in understanding plant responses to water deficit stress and identifying heritable traits for improved drought resilience [5,6].

Numerous reviews have highlighted the detrimental effects of drought stress on plant growth and development, as well as the complex stress-adaptive responses [7,8,9,10]. For example, inadequate water supply negatively affects various morpho-physiological and biochemical processes, including plant growth, reproduction, photosynthesis, respiration, and nutrient uptake and assimilation [11]. The disruption of normal cell metabolism may result in the overproduction and accumulation of reactive oxygen species (ROS) leading to oxidative stress. Oxidative stress, in turn, disrupts the structure and function of lipids, proteins, nucleic acids, and cell membranes, further compromising cellular homeostasis [12,13].

Plants utilize diverse mechanisms to mitigate the detrimental effects of water deficits on cell structure and function. Some plant genotypes avoid drought by enhancing their water-foraging capacity through extensive root systems or by reducing transpiration-dependent water loss via leaf rolling, thick cuticles, and increased stomatal control of transpiration [11]. Conversely, drought tolerance mechanisms enable plants to maintain normal physiological and metabolic functions under water deficit stress [11,14]. Such adaptive responses result from complex processes of stress perception, signal transduction, and alterations in gene expression aimed at restoring cellular homeostasis for survival [15,16]. Some drought-induced physiological and metabolic responses are modulated by the stress hormone abscisic acid (ABA), which accumulates in leaves and roots upon exposure to drought [17].

ABA-dependent and ABA-independent response pathways have been extensively described [18,19] and contribute towards drought tolerance by promoting the production of proteins and metabolites with regulatory and protective functions [15]. For instance, drought-induced metabolites, including sugars, proline, and glycine betaine, participate in osmotic adjustment and protective roles against the osmotic and oxidative effects of water scarcity [20,21]. Likewise, increased enzymatic and non-enzymatic antioxidant activities in plants under drought stress alleviate oxidative cell damage [13,22]. Nevertheless, the efficiency of these adaptive responses may vary depending on the plant species, genotype or developmental stage, and the duration and severity of the prevailing stress [23].

Wheat (*Triticum aestivum*) is one of the most widely grown and consumed cereals globally [24,25]. However, its production yields are negatively affected by drought stress [26,27]. In addition, the wheat germplasm is genetically diverse [28,29] and is grown under equally diverse climatic conditions, worldwide [30,31]. Therefore, comparative studies of wheat varieties with contrasting drought phenotypes are required for deeper insights into the crop’s response to drought stress. Such studies could assist in identifying target genes for developing more drought-resistant wheat genotypes for improved food security. Comparative analyses of physiological and biochemical [32,33,34,35,36,37], transcriptomic [34,38,39], proteomic [37,40,41], and metabolomic [39,41,42] responses of wheat plants to drought stress have been reported. Results of such studies suggest that drought-tolerant wheat genotypes possess greater capacity to maintain cell structure and function, by increasing membrane stability, osmoregulation, and cell redox homeostasis under water-scarce conditions than their drought-sensitive counterparts [32,33,35,36,37,38,40,41,42]. Furthermore, systems biology studies [7,43,44] that integrate plant physiological analyses with molecular cell biochemistry using “omics” technologies are pivotal in unraveling the complex networks of adaptive responses of cells, organelles, and whole plants to drought. Some of the identified drought-responsive genes have been functionally validated using transgenic plants [45,46] and serve as promising candidates for improving the drought resilience of food crops [47].

Therefore, this study investigated the growth, physiological, biochemical, and leaf proteome responses of two contrasting wheat cultivars to drought stress. We hypothesized that contrasting wheat varieties utilize networks of divergent plant responses to water deficit stress, which ultimately determine their drought phenotypes.

## 2. Results

### 2.1. Growth and Physiological Responses of BW4074 and BW35695 Wheat Varieties towards Drought Stress

**Figure 1:**
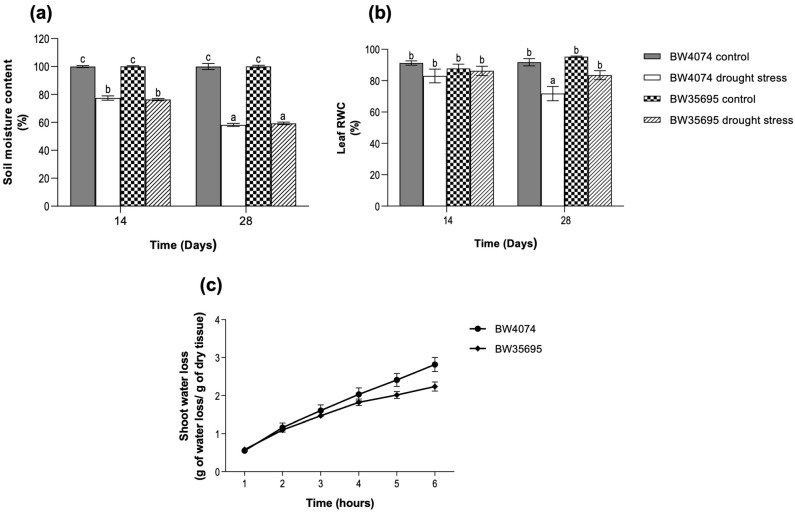
Soil moisture content, relative water content, and shoot water loss in potted wheat plants exposed to drought stress treatment. (**a**) Soil moisture content (*n* = 3); (**b**) leaf relative water content (RWC; *n* = 4); (**c**) relative shoot water loss (*n* = 5). Two-week old plants of BW4074 (drought-susceptible) and BW35695 (drought-tolerant) wheat varieties were exposed to drought stress by withholding water for 28 days and samples were harvested at the indicated time points for the respective measurements. Data presented as mean ± SE. Different letters indicate significant differences between means at (*p* ≤ 0.05) according to ANOVA and Tukey–Kramer test.

When BW4074 (drought-susceptible) and BW35695 (drought-tolerant) wheat seedlings were two weeks old, watering was stopped for 28 days to induce drought stress. During this period, the extent of soil drying in the water-deprived pots was estimated gravimetrically and presented as a percentage relative to the well-watered controls. The results showed that withholding water from the drought-treatment pots progressively dried the soil with time (Figure 1a). At 14 and 28 days after watering was stopped, the water-deprived soil contained between 76 and 77 and 53 and 59% of moisture relative to the well-watered controls, respectively (Figure 1a). However, there was no significant difference in soil moisture content between the two wheat varieties at each time point, suggesting comparable levels of water deficit stress.

We observed a decline in the leaf relative water content (RWC) of both wheat varieties only after 28 days of no watering (Figure 1b). However, the decrease was only statistically significant in the drought-susceptible variety BW4074 (Figure 1b). We also assessed the rate of water loss from detached shoots of both wheat varieties as a proxy for the plants’ capacity to retain water under dehydration stress. The results indicated that the rate of water loss was not statistically different between the two varieties; however, BW4074 was more likely to lose water faster than BW35695 (Figure 1c). Plant growth measurements taken on day 28 of the drought stress treatment showed no significant differences in all parameters measured compared to the controls, except for the decline in shoot fresh weight observed in BW35695 (Figure S1). Taken together, our results suggest that upon exposure to similar levels of soil moisture deficit stress, the drought-tolerant wheat variety BW35695 has a greater water retention capacity compared to the drought-susceptible BW4074.

### 2.2. Drought Stress Increases the Levels of Photosynthetic Pigments Mainly in the Drought-Tolerant Wheat Variety

**Figure 2:**
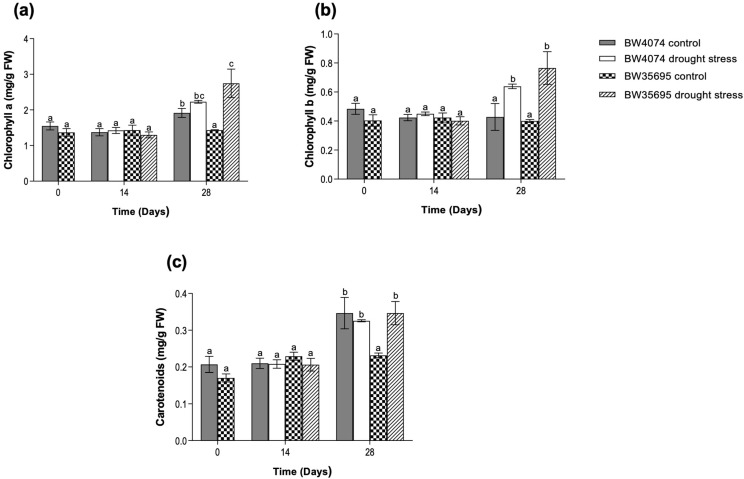
Effects of drought stress on chlorophyll and carotenoid content of wheat. (**a**) Chlorophyll A; (**b**) chlorophyll B; (**c**) carotenoid content. Two-week old plants of BW4074 (drought-susceptible) and BW35695 (drought-tolerant) wheat varieties were exposed to drought stress by withholding water for 28 days and samples were harvested at the indicated time points for the respective measurements. Data presented as mean ± SE (*n* = 3). Different letters indicate significant differences between means at (*p* ≤ 0.05) according to ANOVA and Tukey–Kramer test.

Chlorophyll A and B and carotenoid contents were evaluated in both wheat varieties during the 28 days of water deprivation. As mentioned in our methods description, chlorophyll and carotenoid content measurements were performed on days 0, 14, and 28 to investigate the effects of different levels of drought stress on the parameters and inform us on the possible harvest times for subsequent experiments on reactive oxygen species (ROS) and antioxidant enzymatic assays, and leaf proteome and gene expression analyses. When watering was stopped on day 0, both wheat varieties had similar amounts of photosynthetic pigments in leaves, and these levels remained unchanged between treatments and varieties on day 14 (Figure 2). However, as the duration of stress progressed to day 28, chlorophyll A and B and carotenoid levels increased by 92, 91, and 50%, respectively, in the water-deprived BW35695 plants relative to the well-watered controls (Figure 2a–c). In the drought-susceptible BW4074 variety, chlorophyll A and carotenoid contents remained unchanged following 28 days of drought stress (Figure 2a,c), while that of chlorophyll B marginally increased by 49% relative to the control (Figure 2b). These results suggest that BW35695 increased the biosynthesis and/or accumulation of photosynthetic pigments under drought conditions.

### 2.3. Drought Stress Induces Oxidative Stress in Wheat Tissues

**Figure 3:**
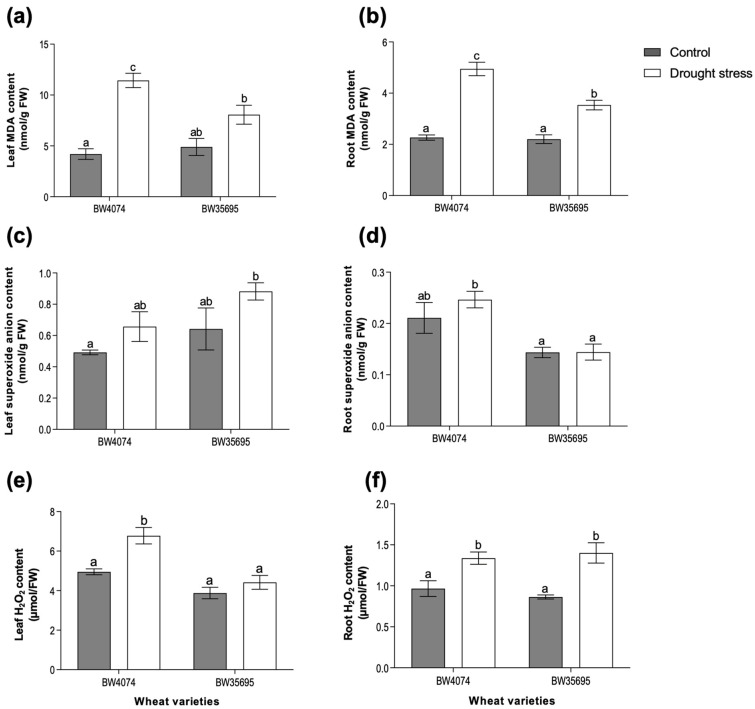
Effects of drought stress on lipid peroxidation and ROS accumulation in wheat plants. Malondialdehyde (MDA) content was used as a proxy for lipid peroxidation, while H_2_O_2_ and superoxide (O_2_^−^) content analyses were used to assess the extent of oxidative stress. Levels of (**a**) leaf MDA; (**b**) root MDA; (**c**) leaf O_2_^−^; (**d**) root O_2_^−^; (**e**) leaf H_2_O_2_; (**f**) root H_2_O_2_. Two-week old plants of BW4074 (drought-susceptible) and BW35695 (drought-tolerant) wheat varieties were exposed to drought stress by withholding water for 28 days for the various measurements. Data presented as mean ± SE (*n* = 3). Different letters indicate significant differences between means at (*p* ≤ 0.05) according to ANOVA and Tukey–Kramer test.

Since oxidative stress is a secondary stress of many environmental factors, including drought, we evaluated lipid peroxidation levels and ROS contents in leaves and roots of both wheat varieties following 28 days of drought stress (Figure 3). In this study, malondialdehyde (MDA) content was used as a proxy for lipid peroxidation, while ROS contents were used to assess the extent of oxidative stress. Compared to their respective well-watered controls, the drought-stressed BW4074 plants exhibited a 118 and 172% increase in leaf and root MDA content, respectively (Figure 3a,b). For the drought-tolerant BW35695, the MDA content increased by a modest 64% and 60% in leaves and roots following drought stress, respectively, and this increase was only statistically significant in the root tissue (Figure 3a,b).

Although an upward trend in leaf superoxide anion (O_2_^−^) content was observed in both wheat varieties following 28 days of drought stress, the increase was not significantly different from the respective controls (Figure 3c). Similarly, the root superoxide content of both varieties remained unchanged following drought stress (Figure 3d). Hydrogen peroxide (H_2_O_2_) content increased by 38% and 62% in the roots of BW4074 and BW35695, respectively, following 28 days of drought stress (Figure 3f). In the leaves, however, only BW4074 drought-stressed plants exhibited a 37% significant increase in H_2_O_2_, while that of BW35695 remained unchanged (Figure 3e). Taken together, results from the lipid peroxidation and ROS content analyses suggest that the 28 days of drought stress induced oxidative stress in both wheat varieties; however, the extent of oxidative damage was more pronounced in the drought-susceptible variety BW4074 compared to BW35695.

### 2.4. Drought-Induced Accumulation of Osmoprotectants in Wheat Leaf and Root Tissues

**Figure 4:**
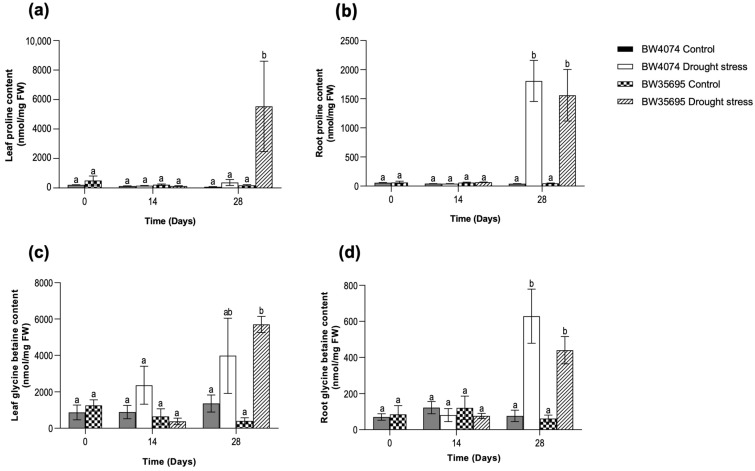
Effects of drought stress on proline and glycine betaine content of wheat plants. Levels of (**a**) leaf proline; (**b**) root proline; (**c**) leaf glycine betaine content; (**d**) root glycine betaine. Two-week old plants of BW4074 (drought-susceptible) and BW35695 (drought-tolerant) wheat varieties were exposed to drought stress by withholding water for 28 days and samples were harvested at the indicated time points for the respective measurements. Data presented as mean ± SE (*n* = 3). Different letters indicate significant differences between means at (*p* ≤ 0.05) according to ANOVA and Tukey–Kramer test.

Leaf and root tissue samples were harvested on days 0, 14, and 28 of drought stress treatment to analyze the differential accumulation of the osmoprotectants, proline and glycine betaine, in both wheat varieties. On days 0 and 14 of the stress treatment, no significant changes in the levels of both osmolytes were observed between treatments within and across the wheat varieties (Figure 4a–d). However, on day 28 of the drought stress treatment, leaf proline levels massively increased only in water-deprived BW35695 plants relative to the control but remained unaltered in the drought-susceptible BW4074 (Figure 4a). In contrast, both proline and glycine betaine accumulated in the leaves and roots of the two wheat varieties on day 28 of drought stress (Figure 4b–d). However, no significant differences were observed in the levels of each osmolyte between similar tissues of the two wheat varieties. We also observed that both osmoprotectants were more abundant in the leaves than the roots (Figure 4). Collectively, our results suggest tissue-specific and varietal differences in the accumulation of proline and glycine betaine under the experimental conditions of this study.

### 2.5. The Drought-Tolerant Wheat Variety BW35695 Possesses Greater Antioxidant Enzyme Activities Compared to Drought-Susceptible BW4074 under Drought Stress

**Figure 5:**
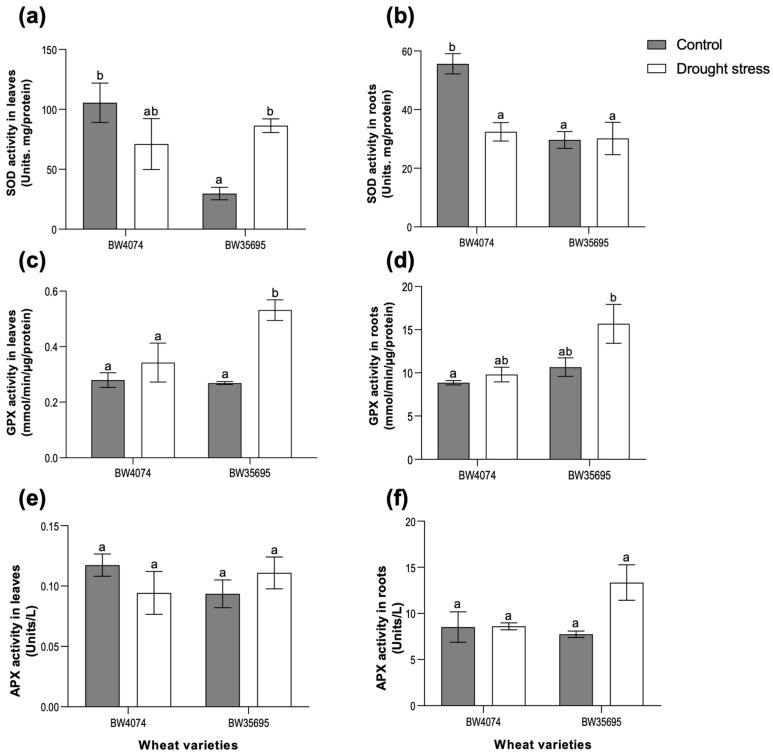
Effects of drought stress on antioxidant enzyme activities of wheat plants. Enzymatic activities of (**a**) leaf superoxide dismutase (SOD); (**b**) root SOD; (**c**) leaf guaiacol peroxidase (GPX); (**d**) root (GPX); (**e**) leaf ascorbate peroxidase (APX); and (**f**) root APX. Two-week old plants of BW4074 (drought-susceptible) and BW35695 (drought-tolerant) wheat varieties were exposed to drought stress by withholding water for 28 days for the various measurements. Data presented as mean ± SE (*n* = 3). Different letters indicate significant differences between means at (*p* ≤ 0.05) according to ANOVA and Tukey–Kramer test.

Following the observed drought-induced oxidative stress and oxidative damage in wheat leaves and roots (Figure 3), we evaluated the antioxidant capacity of the plants to scavenge for ROS. This was achieved by measuring the activities of selected antioxidant enzymes in the leaf and root tissues of both wheat varieties following 28 days of drought stress (Figure 5). A 191% increase in the activity of superoxide dismutase (SOD) was observed in BW35695 drought-stressed leaves relative to the controls (Figure 5a), while that of the roots remained unchanged (Figure 5b). Conversely, a downward trend in SOD activity was observed in the BW4074 drought-stressed plant leaves and roots relative to the controls, with a 42% significant decrease observed only in the roots (Figure 5a,b).

Guaiacol peroxidase (GPX) activity increased by 98% in the leaf tissue of BW35695 following 28 days of drought stress, while that of BW4074 remained unaltered (Figure 5c). Similarly, the root GPX activity of drought-stressed BW4074 plants remained unchanged relative to the controls, while a non-significant increase was observed for the drought-tolerant BW35695 (Figure 5d). Ascorbate peroxidase (APX) activities of the leaves and roots remained largely unchanged following 28 days of drought stress treatment in both wheat varieties, except for a 72% non-significant increase in BW35695 (Figure 5e–f). Our results suggest that the drought-tolerant wheat cultivar BW35695 possesses greater enzymatic antioxidant capacity to scavenge ROS and limit oxidative stress damage under conditions of water scarcity compared to the drought-susceptible BW4074.

### 2.6. The Drought-Responsive Leaf Proteome of Two Contrasting Wheat Varieties

#### 2.6.1. Drought Stress Modulates the Accumulation of Total Soluble Leaf Proteins of Wheat

We complemented the drought-induced physiological and biochemical analyses of the two contrasting wheat varieties with a gel-free leaf proteomic investigation under the same drought conditions. For this experiment, we used the isobaric tags for relative and absolute quantification (iTRAQ) method in combination with liquid chromatography-tandem mass spectrometry (LC-MS/MS) to identify the drought-responsive wheat leaf proteins following 28 days of no watering. In this study, proteins with at least one sequenced peptide were regarded as positively identified, resulting in 1062 and 882 positively identified leaf proteins of BW4074 and BW35695, respectively. The full peptide data are given in Tables S2 and S3. A Student’s *t*-test at *p* ≤ 0.05 was used to analyze the iTRAQ data for differential protein expression patterns between the control and drought-stressed treatment groups of each wheat variety.

**Figure 6:**
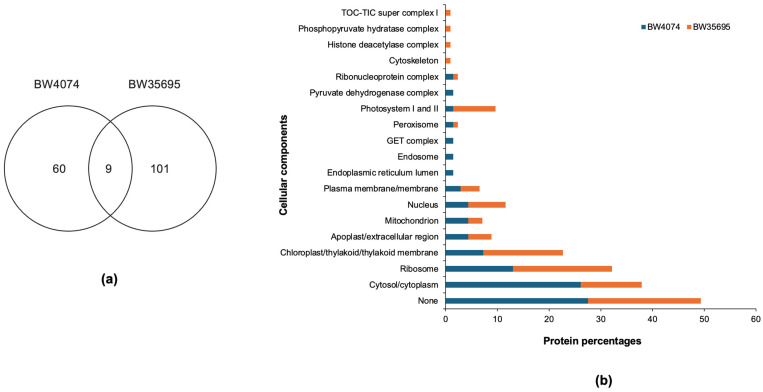
Total number of drought-responsive leaf proteins of two wheat varieties and their putative cellular locations. (**a**) A Venn diagram of the drought-responsive leaf proteins of two wheat varieties. (**b**) Gene ontology terms for cellular locations retrieved from the UniProt database. BW4074 is drought susceptible, while BW35695 is drought tolerant.

For the drought-susceptible BW4074 variety, 69 of the 1062 proteins were differentially expressed in response to drought stress. Of these BW4074 leaf proteins, 41 (59%) were upregulated while 28 (41%) were downregulated. For the drought-tolerant BW35695 variety, 110 leaf proteins were drought responsive, of which 58 (52%) were upregulated while 52 (48%) were downregulated. A summary of this proteome data is given in Table 1, while the full iTRAQ quantitation data are given in Tables S4 and S5 for BW4074 and BW35695, respectively. We also found nine drought-responsive proteins that were common between the two wheat varieties, while 60 and 101 proteins were unique to BW4074 and BW35695, respectively (Figure 6a).

We then retrieved Gene Ontology (GO) data and protein family names of the drought-responsive proteins from the UniProt and InterPro databases and used this information to functionally group the proteins. The GO terms, protein family names, and the putative functional categories of the drought-responsive proteins are summarized in Tables S6 and S7 for BW4074 and BW35695, respectively. Due to the extensive sizes of Tables S6 and S7, we generated Table 2 and Table 3 as shortened protein lists for illustrative purposes. The drought-responsive proteins in Table 2 and Table 3 are those with a minimum fold change of 1.5; however, all results presentations and discussions in this study are based on the full lists of drought-responsive proteins in Tables S6 and S7.

The GO cell localization data show that the drought stress treatment affected the accumulation of leaf proteins present in various cellular locations, irrespective of the wheat variety (Figure 6b). Some of the highly represented cellular locations common to both wheat proteomes include the cytoplasm/cytosol, ribosome, chloroplast, Photosystem I and II, mitochondrion, and the nucleus (Figure 6b). Likewise, the drought-responsive proteins are potentially involved in diverse biological processes (Figure S2) and molecular functions (Figure S3). Biological process terms of ‘photosynthesis’ and ‘translation’ were highly represented in the proteome of the drought-tolerant variety BW35695 while ‘translation’ dominated the BW4074 proteome. Consistent with the biological process terms, molecular functional terms of ‘chlorophyll binding’ and ‘structural constituent of ribosome’ were highly represented in BW35695, while ‘structural constituent of ribosome’ emerged as the most represented molecular functional term for the drought-sensitive BW4074. Overall, our GO analyses data demonstrate that drought stress modulates the accumulation of proteins in various cellular locations and is involved in diverse biological processes. Both wheat varieties seemed to have largely reprogrammed translation-related processes, while the drought-tolerant variety BW35695 also reconfigured various biochemical aspects of photosynthesis (Figures S2 and S3).

#### 2.6.2. Drought-Responsive Wheat Leaf Proteins Are Implicated in Diverse Functional Roles

**Figure 7:**
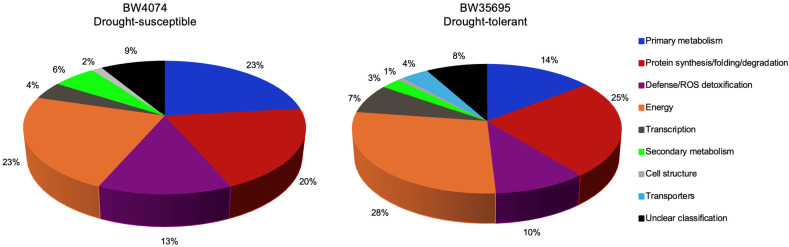
Putative functional groups of the drought stress-responsive leaf proteins of wheat.

To understand the functional roles of the identified differentially expressed leaf proteins in drought response, we used the GO data and protein family names (Tables S6 and S7) to group proteins into theoretical functional categories (Tables S6 and S7; Figure 7). For this task, we mainly followed the classification scheme suggested by Bevan et al. [48]. The 110 drought-responsive leaf proteins of BW35695 had putative functions in energy (28%), protein synthesis/folding/degradation (25%), primary metabolism (14%), defense/ROS detoxification (10%), transcription (7%), transporters (4%), secondary metabolism (3%), and cell structure (1%). Likewise, the 69 drought-responsive leaf proteins of BW4074 had putative functions in primary metabolism (23%), energy (23%), protein synthesis/folding/degradation (20%), defense/ROS detoxification (13%), transcription (4%), secondary metabolism (6%), and cell structure (2%). However, 9% and 8% of the differentially expressed proteins for BW4074 and BW35695 had limited GO data to facilitate their functional grouping and thus remained unclassified (Tables S6 and S7; Figure 7). The transporters’ functional group with four (4%) proteins was only present in the proteome of BW35695 (Figure 7).

**Figure 8:**
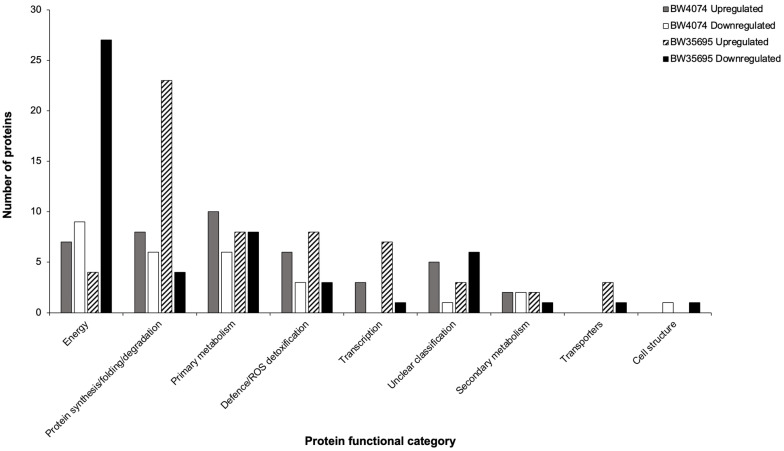
Number of up- and downregulated leaf proteins per functional group for each wheat variety under drought stress. BW4074 is drought susceptible, while BW35695 is drought tolerant.

The distribution patterns of up and downregulated proteins of each functional group per wheat variety are shown in Figure 8. Although the absolute counts of up- and downregulated proteins varied between functional group across the two wheat varieties, we noted comparable trends in some functional catagories. For example, (i) there were generally more upregulated proteins in the defense/ROS detoxification, protein synthesis/folding/degradation, and transcription functional groups of both wheat varieties; (ii) most energy-related proteins were downregulated in both varieties; and (iii) diverse transporters were only identified in BW35695 and most were upregulated (Figure 8). These results suggest that wheat plants under drought stress conditions reprogram various physiological and biochemical processes, and some of these changes may be similar, dissimilar, or even unique between varieties with contrasting drought phenotypes. Brief results presentations of the main trends in selected functional groups are outlined below.

##### Drought Stress Largely Downregulates Energy-Related Proteins, Particularly Those Associated with Photosynthesis

The energy group constituted the largest functional category in BW35695 with 31 (28%) drought-responsive proteins (Table S7, Figure 7). Of these proteins, 27 (87%) were downregulated and mostly involved in photosynthesis and ATP synthesis (Table S7; Figure 8). For example, three ATP synthase subunits involved in the production of ATP, and 18 chlorophyll-binding proteins of Photosystems (PS) I and II, a large subunit of ribulose biphosphate carboxylase (Rubisco), and an oxygen-evolving enhancer protein, all with core functions in photosynthesis, were downregulated (Table S7). Conversely, aconitate hydratase with accession A0A3B6QKY1 was the most upregulated protein with a 2.05 fold change, while a cytochrome b6/f complex protein with accession A0A3B6U9Q7 was the most downregulated by a fold change of −3.44 (Table S7). For BW4074, 9 of the 16 (56%) energy-related proteins were downregulated; however, no prominent trends were observed in this protein list. Nevertheless, an ATP synthase subunit and photosynthesis-related proteins such as the ferrodoxin-NADP reductase and Photosystem I iron-sulfur center protein were downregulated. Collectively, our results suggest that wheat plants, in particular the drought-tolerant BW35695 variety, respond to dehydration stress by downregulating proteins involved in photosynthesis and ATP production.

##### Drought Stress Upregulates Proteins Involved in Defense Response, Protein Homeostasis, and Transcription

The transcription functional group consisted of three (4%) drought-responsive proteins in BW4074 and all were upregulated (Table S6, Figure 7 and Figure 8). Similarly, the drought-tolerant BW35695 variety had eight (7%) transcription-related proteins and seven were upregulated (Table S7, Figure 7 and Figure 8). Collectively, the upregulated proteins in both wheat varieties were histones of types H2A and H2B, linker histones H1/H5, and various regulators of transcription within the STI1 domain-containing protein, RNA binding protein HABP4/SERBP1-like, methyl-CpG binding domain-containing protein 10/11, and the high-mobility group protein HMGA families (Tables S6 and S7). The only downregulated protein in BW35695 was another histone H2A isoform named protein H2A.7 with accession Q43312 (Table S7).

The protein synthesis/folding/degradation functional category consisted of 14 (20%) drought-responsive proteins in BW4074 and 27 (25%) for BW35695 (Figure 7). Of these proteins, 8 and 23 were upregulated in BW4074 and BW35695, respectively, while the rest were downregulated (Tables S6 and S7; Figure 8). Most of the small and large ribosomal subunit proteins involved in translation, amounting to five in BW4074 and 18 in BW35695, were upregulated. Examples of downregulated proteins include those with protein folding/refolding functions such as peptidyl-prolyl cis-trans isomerase (accession Q93XQ6) and a trigger factor ribosomal binding protein (accession W5D1B3) in BW4074, and chaperonin CP60-2 with accession A0A3B6JPZ3 in BW35695. Only two proteolysis-related proteins were identified in this study and both were found in the BW35695 leaf proteome (Table S7). Of these two proteins, the ATP-dependent zinc metalloprotease (accession A0A3B6TR29) was downregulated, while a dipeptiylpeptidase IV N-terminal domain-containing protein (accession A0A3B6HYY4) with serine-type peptidase activity was upregulated (Table S7).

The defense/ROS detoxification functional group consitituted 13% of the drought-responsive proteins of BW4074 with nine proteins (Table S6; Figure 7). The same group made up 10% of the drought-responsive proteome of BW35695 with 11 proteins (Table S7; Figure 7). Of these proteins, six and eight were upregulated in BW4074 and BW35695, respectively (Figure 8). Antioxidant enzymes such as SOD, gluthathione reductase (GR), and monodehydroascorbate reductase (MDAR) were upregulated in BW4074, while a secretory peroxidase was downregulated (Table S6). Other upregulated proteins in the drought-susceptible variety include a cold-induced 16 protein which regulates ABA biosynthesis and a late embrogenesis abundant (LEA) protein, while a putative protein disulphide isomerase-like (accession D8L9B5) which responds to ER stress was the most downregulated protein with a −2.57 fold change (Table S6). Similar trends were observed in the drought-tolerant BW35695 where a cold-responsive LEA-RAB-related COR protein with accession A0A172WCB1 and an uncharacterized nodulin-related protein with accession A0A3B6EFA0 were the two most upregulated proteins with fold changes of 3.23 and 2.66, respectively (Table S7). Taken together, these results suggest that wheat plants under drought stress reprogram transcriptional activities, resulting in increased protein synthesis, and the accumulation of various proteins involved in defense response, regulation of ABA biosynthesis, ROS detoxification, and other responses to water deficit stress.

##### Other Functional Categories of Drought-Responsive Wheat Leaf Proteins

Other drought-responsive proteins identified in this study were categorized into theoretical functional groups of primary metabolism, secondary metabolism, cell structure, and transporters (Tables S6 and S7; Figure 7). Although the primary metabolism group consitituted a large proportion of the drought-responsive proteomes of both wheat varieties (Figure 7), there were no striking trends emerging from these data, except that various proteins involved in the metabolism of fatty acids, amino acids, phosphates, polysaccharides, and nucleosides were modulated (Tables S6 and S7). In the drought-sensitive BW4074 variety, a delta-1-pyrroline-5-carboxylate synthase (accession W5ACM8) involved in proline biosynthesis was upregulated with a fold change of 1.52 (Table S6). In BW35695, a beta-glucosidase (accession A0A3B6KPK9) belonging to the cellulose degradation glycosyl hydrolase 3 family was highly upregulated by a factor of 2.27 (Table S7). In the secondary metabolism group, a zeta-carotene desaturase (accession A0A3B6C785) was involved in the biosynthesis of carotenoids, and an AB hydrolase-1 domain-containing protein (accession A0A3B6DPV0), belonging to the epoxide hydrolase-like protein family were both downregulated in BW4074 (Table S6).

Furthermore, in BW35695, a delta-aminolevulinic acid dehydratase (accession A0A3B6QDX1) involved in chlorophyll biosynthesis was also downregulated. An actin protein (accession W5FAY5) identified in BW35695 and a protein curvature thylakoid I-related protein (accession A0A3B6SEK0) identified in BW4074 were both downregulated, further illustrating the negative effects of drought on the strutural intergrity of the cytoskeleton and the chloroplast thylakoid membrane (Tables S6 and S7). The BW35695 drought-responsive leaf proteome also contained transporter proteins such as a porin-related uncharacterized protein, a lipid-transporting non-specific lipid transfer protein, a chloroplast inner envelope protein, and an STI1/HOP DP-containing protein both with putative roles in protein transport or targeting into the chloroplast (Table S7). All these proteins, except the chloroplast inner envelope protein, were upregulated, indicating that the drought-tolerant wheat variety also reconfigures various intracellular transport systems in response to drought. Although no particular transporter was identified in the BW4074, the anion-transporting ATPase-like domain-containing protein grouped under protein synthesis due to its asssociated role with translation has a putative function in the post-translational protein targeting to the ER membrane (Table S6). Together with the unclassified proteins listed in Tables S6 and S7, our wheat leaf proteome results illustrate a vast array of cellular processes that are modulated by dehydration stress.

##### Common Drought-Responsive Proteins between BW4074 and BW35695

Following our observation that nine of the differentially expressed proteins of this study are common between the two wheat varieties (Figure 6a), we statistically analyzed the fold changes of each protein between the two varieties (Table 4). The data show that five of these common proteins with accessions A0A3B6TJK6, A0A3B6MJZ2, A0A3B6MTE3, A0A3B6QKY1, and A0A3B6MQA1 had comparable upward or downward fold changes in response to the drought stress treatment. However, a fibronectin type III-like domain-containing protein with accession A0A1D5URN5, belonging to the beta-D-xylosidase protein family, was more downregulated in the drought-sensitive BW4074 than BW35695. Conversely, a Photosystem I iron-sulfur center protein (accession P69415) which functions in photosynthesis was more downregulated in the drought-tolerant wheat variety than in BW4074. Contrasting expression patterns were also observed for the 30S ribosomal protein S20 (accession W5D1D3), which was downregulated in BW4074 but upregulated in BW35695. Likewise, an unclassified RRM domain-containing protein (accession A0A3B6MQA1) with eukaryotic RNA-binding properties was upregulated in BW4074 but downregulated in BW35695. Overall, the observed similarities and differences in expression trends of these common stress-responsive proteins illustrate the complex dynamics in stress responses to drought between varieties of a single species with contrasting phenotypes. Some of these common changes may represent fundamental stress responses required by plants during exposure to unfavorable conditions, while contrasting expression patterns of the same protein isoform in the two wheat varieties possibly highlight key biological processes that may determine the probability of plant survival under stress conditions.

### 2.7. Pathway Enrichment Analysis and Protein–Protein Interactions

#### 2.7.1. KEGG Pathway Enrichment Analysis

**Figure 9:**
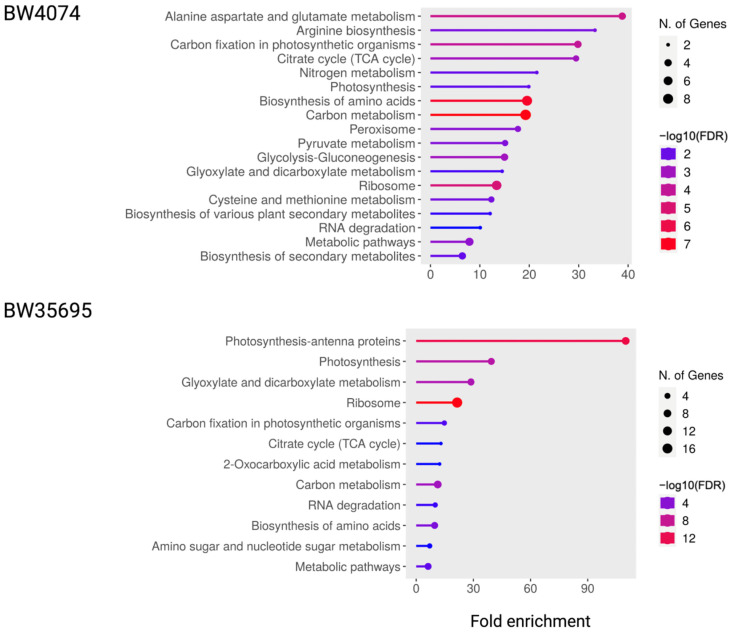
KEGG pathway enrichment analysis of drought-responsive leaf proteins of wheat. BW4074 is drought susceptible, while BW35695 is drought tolerant. The significance of the KEGG path is based on the Student’s *t*-test (*p* ≤ 0.05). The sizes of dots represent the number of genes in each pathway at a scale indicated by the legend within the graphics. To correct for multiple testing, False Discovery Rate (FDR) was calculated using the Benjamini–Hochberg method, and the measure of FDR is indicated by the colors at the logarithmic scale provided in the legend within the graphics.

To analyze the functions of the drought-responsive leaf proteins of the two wheat varieties, a Kyoto Encyclopedia of Genes and Genomes (KEGG) pathway enrichment analysis was conducted (Figure 9). Ten of the identified pathways were present in both varieties. These include citrate cycle (TCA cycle), carbon fixation in photosynthetic organisms, photosynthesis, glyoxylate and dicarboxylate metabolism, carbon metabolism, biosynthesis of amino acids, ribosome, and metabolic pathways. Pathways exclusive to BW4074 were alanine aspartate and glutamate metabolism, arginine biosynthesis, nitrogen metabolism, peroxisome, pyruvate metabolism, cysteine and methionine metabolism, glycolysis-gluconeogenesis, biosynthesis of secondary metabolites, and biosynthesis of various plant secondary metabolites. Conversely, pathways unique to BW35695 were photosynthesis-antenna proteins, 2-Oxocarboxylic acid metabolism, RNA degradation, and amino sugar and nucleotide sugar metabolism. The top two most significantly enriched pathways in BW4074 were associated with alanine aspartate and glutamate metabolism and arginine biosynthesis whilst those in BW35695 were related to photosynthesis-antennae and photosynthesis (Figure 9). Results of our KEGG pathway enrichment analysis further complement the putative functional groupings data (Figure 7), which suggest the drought stress modulates various biological processes in wheat leaves, but mainly primary metabolism in BW4074 and photosynthesis in BW35695 (Tables S6 and S7).

#### 2.7.2. Protein–Protein Interaction Analysis

**Figure 10:**
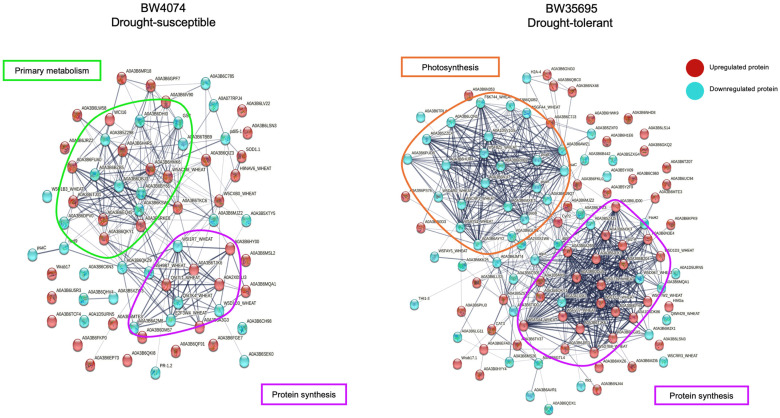
Protein–protein interactions analysis of BW4074 and BW35695 drought-responsive leaf protein using the STRING database. In this network, nodes are proteins and lines represent functional associations between proteins. Nodes in red are upregulated proteins while nodes in blue are downregulated proteins. Thicker lines represent stronger associations between the interacting proteins.

To identify the interactions between drought-responsive leaf proteins of BW4074 and BW35695 wheat varieties, a protein–protein interaction analysis was conducted using the STRING (Search Tool for the Retrieval of Interacting Genes/Proteins) database. Primary metabolism and protein synthesis were the two main interacting biological processes for BW4074, whilst photosynthesis and protein synthesis emerged as the two main interacting functional groups for BW35695 (Figure 10). The abundance of the leaf proteins involved in the photosynthesis functional group decreased following drought stress treatment as indicated by blue nodes. In the protein synthesis category, the majority of the proteins were upregulated in response to drought stress as represented by red nodes, and only two ribosomal proteins, A0A341U912 and W5D067, were downregulated. Overall, the protein–protein interaction maps suggest wheat plants under drought stress modulate various biological processes by altering the expression levels of proteins. These stress-responsive proteins associate with each other to varying degrees to coordinate the desired response mechanism; such protein interactions may also differ between wheat plants of contrasting drought phenotypes.

### 2.8. Gene Expression Analysis Using qRT-PCR

**Figure 11:**
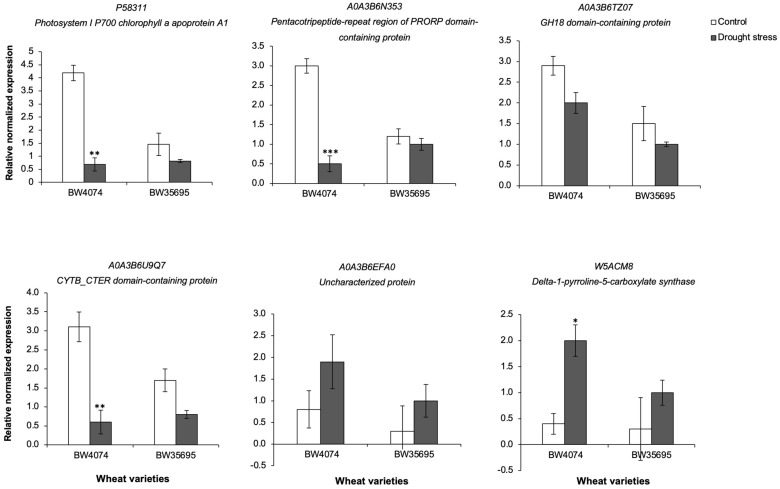
Gene expression analysis in wheat leaf tissue following drought stress treatment. Drought-susceptible BW4074 and drought-tolerant BW35695 wheat plants were exposed to drought stress by withholding water for 28 days and gene expression analysis was performed using qRT-PCR. Data presented as the mean ± SE (*n* = 3). ** and *** represent statistical significance between the control and drought-stressed samples at *p* ≤ 0.05, 0.01, and 0.001, respectively, using a Student’s *t*-test on the CFX Maestro software version 4.1.2433.1219.

We selected some targets for analysis at the gene expression level in leaf tissues on both wheat varieties following 28 days of drought stress (Table S1). The six target genes were randomly selected from the lists of differentially expressed proteins in the two wheat varieties (Tables S6 and S7). Our results show that qRT-PCR data validated the protein data for some targets. For example, gene expression results show that the *delta-1-pyrroline-5-carboxylate synthase* (*W5ACM8*) gene was upregulated by a factor of two in BW4074 following drought stress treatment (Figure 11), which corresponded with the iTRAQ result showing that its protein with accession W5ACM8 was upregulated by a factor of 1.52 in the same wheat variety (Table S6).

For the rest of the targets (Figure 11), the qRT-PCR data revealed that differential expression is either controlled post-transcriptionally or that the time points where transcript abundance aligns with protein abundance are different. For instance, the iTRAQ data showed that the Photosystem I P700 chlorophyll A apoprotein A1 with accession P58311 and the CYTB_CTER domain-containing protein with accession A0A3B6U9Q7 were highly downregulated in BW35695 (Table S7), but not listed among the drought-responsive proteins of BW4074 (Table S6). Nonetheless, gene expression data revealed that both genes, *P58311* and *A0A3B6U9Q7*, were downregulated in both wheat varieties but were only statistically significant in the drought-sensitive BW4074 (Figure 11). Similarly, the *GH18 domain-containing protein* (*A0A3B6TZ07*) and the *Uncharacterized protein* (*A0A3B6EFA0*) genes did not show any significant change in expression following the drought treatment in both wheat varieties (Figure 11), yet their corresponding proteins were highly upregulated in BW35695 during drought stress (Table S7). Overall, our qRT-PCR analysis suggests that the drought-induced upregulation of the delta-1-pyrroline-5-carboxylate synthase (W5ACM8) protein observed in BW4074 (Table S6) is transcriptionally regulated under the experimental conditions of this study. The other five protein targets used in gene expression (Figure 11) could either be post-transcriptionally regulated or the abundances of the proteins and transcripts differentially accumulate at different time points.

## 3. Discussion

We used the drought-susceptible BW4074 and drought-tolerant BW35695 wheat varieties to comparatively investigate the differential effects of drought stress on the growth, physiology, biochemistry, and leaf proteome expression of wheat plants. Our results showed that withholding water supply to potted wheat plants for 28 days resulted in progressively drying soils (Figure 1a), equivalent to mild and moderate drought stress conditions [49,50], on days 14 and 28 of the stress treatment, respectively. The intensity of water deficit stress at each time point was not significantly different between the two wheat varieties (Figure 1a), suggesting that our experimental system could be used to compare the differential responses of the wheat varieties to drought. Consequently, any differences in physiological and/or molecular responses between the two wheat varieties could be attributed to the genetic constitution and drought phenotypes of the plants, rather than variations in the levels of water deficit stress imposed.

The leaf relative water content (RWC) and shoot water loss results (Figure 1b,c) suggest that the drought-tolerant variety BW35695 possesses greater water retention capacity under dehydration stress than the drought-susceptible BW4074. Similar findings have been reported in drought-stressed wheat (*Triticum aestivum*) [37,51], sorghum (*Sorghum bicolor*) [52], and soybean (*Glycine max*) [53] plants under drought conditions, where the drought-tolerant genotypes remained more hydrated than their drought-sensitive counterparts. A higher plant tissue water content under drought conditions contributes towards optimal cellular hydration, and the preservation of cell structure and function [14]. However, more studies are required to evaluate the contributions of stomatal and/or non-stomatal responses [32,54] of BW4074 and BW35695 in water retention processes under drought stress.

Although the 28 days of moderate drought stress treatment did not cause elaborate changes in the measured growth parameters in both wheat varieties (Figure S1), the chlorophyll A and b and carotenoid levels increased in BW35695 (Figure 2). Conversely, only chlorophyll B marginally increased in BW4074 relative to the controls (Figure 2). Chlorophyll A and B and carotenoids are essential light-harvesting molecules of photosynthesis [55,56] and their increased accumulation under drought conditions may enhance the photosynthetic potential of plants for increased growth and survival. In contrast to our results, another comparative study between drought-tolerant and drought-susceptible wheat varieties revealed a decline in chlorophyll A and B content of both varieties following drought stress [51]. However, it is unclear whether the increased levels of chlorophyll A and B in the BW35695 wheat variety (Figure 2) are due to increased biosynthesis and/or reduced drought-induced degradation of the pigments. Nevertheless, we suggest more physiological measurements of other parameters including the chlorophyll index and photosynthesis rate [32,41,54] to understand the differential effects of drought on pigment content, photosynthesis, and yield components of wheat.

Dehydration stress disrupts normal cell metabolism which may result in increased production of reactive oxygen species (ROS). Any imbalances between ROS hyperaccumulation and their detoxification may lead to oxidative stress damage of cell components [13,57]. In our study, the 28 days of drought stress caused increased lipid peroxidation in BW4074 plant tissues (Figure 3a,b), possibly due to increased ROS accumulation (Figure 3c–f), coupled with a reduced enzymatic antioxidant capacity (Figure 5). Conversely, the drought-tolerant BW35695 had lower ROS tissue content, better enzymatic antioxidant activities, and thus reduced membrane damage (Figure 3 and Figure 5). These results further underscore the superior drought-tolerant nature of BW35695 compared to BW4074.

Some drought-induced metabolites possess various regulatory and protective functions in plant cells [15]. For example, apart from their light-harvesting roles during photosynthesis, carotenoids may also function as non-enzymatic antioxidants [57]. Proline and glycine betaine act as osmolytes and osmoprotectants under water-limiting conditions [58,59,60]. Therefore, the observed increase in the level of carotenoids (Figure 2c) and proline (Figure 4a) in the leaves of BW35695 may signify greater potential for osmotic adjustment and ROS scavenging capacity, which could have contributed towards higher RWC (Figure 1b) and lower lipid peroxidation (Figure 3a). The significant increase in the levels of root proline, as well as root and leaf glycine betaine in both wheat varieties following 28 days of stress (Figure 4), also signifies the critical roles of these metabolites in drought response. As reported in other studies of wheat [51], maize (*Zea mays*) [61], and rice (*Oryza sativa*) [62], under drought conditions, we observed higher levels of these osmoprotectants in leaves than roots (Figure 4). However, the reasons behind these tissue-specific differences in metabolite content are unclear from the results of our current study.

We complemented the physiological and biochemical analyses with a leaf proteome study of the two wheat varieties following 28 days of drought stress (Table 1). Our proteome data suggest that the imposed drought stress modulated the levels of proteins involved in various plant biological processes including primary and secondary metabolism, photosynthesis, ATP synthesis, transcription, protein synthesis and homeostasis, ROS detoxification, and other defense-related systems (Tables S6 and S7; Figure 7). We have chosen to focus our discussion on the key common and unique trends observed in the two wheat varieties, highlighting their potential significance in drought response. Understandably so, our proteomics discussion is largely general and speculative in nature, until functional validation studies are performed for the identified drought-responsive proteins and their isoforms.

Our results suggest that both wheat varieties modulated the accumulation profiles of transcription-related proteins, with a marked increase in histones, linker histones, and other regulators of transcription particularly in the drought-tolerant BW35695 variety (Tables S6 and S7; Figure 7 and Figure 8). Histones are involved in chromosomal DNA packing [63], and their upregulation during stress response facilitates transcriptional regulation of stress-responsive genes [64]. Similar results have been reported in other comparative proteome studies of wheat leaves [65], sorghum roots [52], as well as transcriptome analyses of wheat leaves and roots [34,38] under drought stress. It is also well established that changes in transcriptional activities under stress conditions allow plants to reprogram their gene expression patterns and ultimately cell metabolism to produce regulatory and protective proteins and metabolites for stress survival [15]. Therefore, the observed upregulation of transcription-related proteins especially in BW35695 potentially highlights its superior ability to quickly respond to the prevailing stress by modulating gene expression patterns to maintain cell homeostasis for survival.

Our proteome data also suggest that the transcription-related protein changes resulted in increased synthesis and accumulation of drought-responsive proteins. This viewpoint is partly supported by the upregulation of numerous ribosomal proteins involved in translation, ROS-detoxifying enzymes, and other defense proteins identified in both wheat varieties (Tables S6 and S7; Figure 8). However, more ribosomal proteins were upregulated in BW35695 than in BW4074 (Figure 8). Furthermore, two peptidyl-prolyl cis-trans isomerases (Q93XQ6 and A0A3B6MTE3) were upregulated in BW35695. In BW4074, A0A3B6MTE3 was also upregulated, while W5H9B7 was downregulated (Table S6). Peptidyl-prolyl cis-trans isomerases function in protein folding activities [66] and have been implicated in drought responses of wheat [40] and maize [67] leaves. The observed contrasting accumulation patterns of peptidyl-prolyl cis-trans isomerases in the current study possibly points towards differential capacities to correctly fold proteins in BW4074 versus BW35695 under drought stress conditions, which may influence the overall structure and functions of drought-responsive proteins.

Contrary to our antioxidant enzymatic activity results, which implied that the drought-tolerant BW35695 had greater ROS scavenging capacity than BW4074 (Figure 5), we identified drought-responsive antioxidant enzymes in both BW4074 and BW35695 (Tables S6 and S7), albeit from different protein families and with varying fold changes. In BW4074, two superoxide dismutase (SOD) isoforms, a glutathione reductase (GR) and a monodehydroascorbate reductase (MDAR), were upregulated (Table S6), while catalase, a thioredoxin domain-containing protein, germin-like protein, and a secretory peroxidase were upregulated in BW35695 (Table S7). It is plausible that the differences in protein families of the identified antioxidant enzymes between the two wheat genotypes reflect genotypic differences in the wheat responses to oxidative stress. Nevertheless, our proteomics data suggest the existence of diverse drought-responsive enzymatic antioxidants in wheat leaves. Furthermore, the two wheat varieties may utilize different types of ROS scavengers to maintain cellular redox homeostasis and these proteins inevitably require different activity assays to study their involvement in stress response. Therefore, future enzymatic activities assays should be performed for a wider pool of enzymes and substrates.

Some transcriptomics studies of soybean [53], maize [68], and sorghum ([69,70,71] under drought stress have suggested that the constitutive expression of transcripts in leaves and/or roots may vary between contrasting genotypes under normal growth conditions. As such, the overall drought responses of such genotypes may be influenced by the basal levels of specific proteins and metabolites of such transcripts. In addition, tissue-specific proteome responses may also be influenced by multi-gene families where different protein isoforms may have different functional roles depending on their cellular location [72]. Therefore, we recommend meta-analyses of “omics” data of various wheat genotypes and related species under control and drought stress conditions to better understand the implication of constitutive gene/protein/metabolite expression in drought-adaptive responses.

The massive downregulation of proteins involved in photosynthesis and ATP production is another noteworthy trend of this study, particularly for the drought-tolerant BW35695 variety (Tables S6 and S7; Figure 8). It is well established that some plants under drought reduce their rate of photosynthesis [47], possibly as a measure to avoid the over- production and accumulation of ROS [73]. Furthermore, stress tolerant genotypes can enhance the levels of energy metabolism enzymes, thereby increasing ATP production in response to drought stress [73]. However, in our study, both photosynthesis-related proteins and those involved in ATP synthesis were generally downregulated (Table S7). Similar results have also been reported in proteomic studies of wheat leaves [40] and cassava (*Manihot aeculenta*) chloroplasts [74], where the drought-tolerant genotypes reduced the accumulation of proteins related to photosynthesis and ATP production. However, it is unclear why the biochemical contents of chlorophyll A and B increased in BW35695 more than in BW4074 (Figure 2), yet the accumulation patterns of a delta-aminolaevulinic acid dehydratase involved in chlorophyll biosynthesis and that of light-harvesting chlorophyll A-B binding proteins were downregulated in the proteome of this wheat variety (Table S7).

The divergent KEGG pathway enrichment results (Figure 9) and equally distinct protein–protein interaction maps (Figure 10) further demonstrate the extent to which the two genotypes differ in response to drought stress. The BW35695 drought-responsive leaf proteome is highly enriched with photosynthesis-antennae and photosynthesis-related pathways as opposed to primary and secondary metabolism-related pathways in BW4074 (Figure 9). In addition, our protein–protein interactomes highlight the overly complex but interconnected networks of proteins and biological processes in wheat leaves under water deficits (Figure 10). As discussed earlier, these protein interaction networks illustrate that the drought-tolerant BW35695 reprograms its cellular metabolic activities by increasing protein synthesis-related proteins possibly to increase the biosynthesis of other stress-responsive proteins.

## 4. Materials and Methods

### 4.1. Plant Materials, Growth Conditions, and Drought Stress Treatments

Two pure wheat (*Triticum aestivum*) lines, BW4074 (an improved cultivar that is drought susceptible) and BW35695 (a drought-tolerant breeder’s line), obtained from the International Maize and Wheat and Improvement Center (CIMMYT) in Mexico were used in this study. The seeds were germinated on moist paper towels in a growth chamber (Model: GC-539DH, Already Enterprise Inc., Tapei, Taiwan) at 18 °C under dark conditions. Twelve six-day-old seedlings of each wheat variety were transplanted into plastic pots with dimensions of 10 cm top diameter × 6.5 cm bottom diameter × 8.5 cm height containing 150 g potting soil (Culterra, Muldersdrift, South Africa), previously saturated with Nitrosol nutrient solution (Envirogreen (Pty) Ltd., Braamfontein, South Africa). About 32 pots were prepared for each wheat variety. The plants were grown for a further 8 days in the growth chamber at 18/15 °C day/night temperatures under a 16 h photoperiod. All plants were well watered during this growth period.

Fourteen-day-old plants were thinned to ten plants per pot, and the pots were split into two groups for each wheat variety. The first group was for the drought stress treatment, where watering was withheld for 28 days. The second group consisted of control plants that were well watered throughout the experiment. On days 14 and 28 of the stress treatment, we collected three biological replicate pots from each treatment group per wheat variety to measure the soil moisture content as a proxy for the level of drought stress. The soil moisture content was estimated using the gravimetric method [75] with minor modifications. For these measurements, plants were harvested and the fresh weights of soil samples were immediately taken, before soil oven-drying at 105 °C for 48 h for dry weight readings. Three biological replicate soil samples were used for each treatment group per wheat variety.

The effect of water deprivation on wheat plants was assessed by measuring various growth, physiological, and biochemical parameters at days 0, 14, and 28 of treatment. In this study, day 0 denotes the time when watering of the two-week old seedlings was stopped, and the treatment group plants received no further watering thereafter. Leaf and root tissues harvested at these time points were stored at −80 °C for use in biochemical, leaf proteomic, and gene expression analyses. Growth, physiological, and biochemical analyses that required freshly harvested tissues were performed immediately after sampling. All experimental procedures performed in this study had a minimum of three biological replicates as described below.

The first sets of measurements such as leaf relative water content and chlorophyll, carotenoid, proline, and glycine betaine content analyses were conducted using plant tissue samples harvested at 0, 14, and 28 days of drought stress. Our results revealed that both wheat varieties mostly showed some changes in these parameters following 28 days of drought stress and not at day 14; hence, subsequent ROS measurements, antioxidant enzymatic activity assays, and protein and gene expression analyses were conducted on tissue samples harvested on day 28 of the drought stress treatment.

### 4.2. Plant Growth Parameters, Relative Water Content, and Relative Shoot Water Loss Measurements

Plant growth parameters, leaf relative water content (RWC), and relative shoot water loss were determined following 28 days of drought stress. The RWC was estimated on freshly harvested whole leaf samples as described previously [76] with minor modifications [52]. Four biological replicates of the third oldest leaf samples were used per treatment per wheat variety.

The shoot water loss experiment was performed following a previously described method [77] and used as a proxy for the plants’ water retention capacity. Briefly, the above-ground shoots were cut off from five biological replicate plants per treatment per variety, and their fresh weights were immediately taken. The shoots were left on a benchtop at room temperature (~25 °C) and re-weighed at 1 h intervals for 6 h. The relative shoot water loss was calculated using the formula described by Rahman et al. [77].

The length and fresh and dry weight measurements of the roots and shoots were determined for the control and drought-stressed plants of each wheat variety as described previously [52]. Five biological replicates were used per treatment for each measurement.

### 4.3. Biochemical Assays

#### 4.3.1. Chlorophyll and Carotenoid Content

Chlorophyll A and B and carotenoids were extracted using a dimethyl sulfoxide (DMSO) extraction method [78]. Three biological replicates were used per treatment per wheat variety for plants harvested on day 0, 14, and 28 following drought stress. Briefly, 100 mg of each frozen leaf tissue sample was mixed with DMSO and incubated in a water bath at 65 °C for 3 h. The samples were pulse vortexed, and 1 mL aliquots of the supernatant were used for absorbance measurements at 480, 649, and 665 nm against a DMSO blank solution. Chlorophyll A and B and carotenoid contents were estimated using a formula described by Wellburn [79].

#### 4.3.2. Lipid Peroxidation, Hydrogen Peroxide, and Superoxide Content

The levels of malondialdehyde (MDA), hydrogen peroxide (H_2_O_2_), and superoxide anion (O_2_^−^) were estimated in leaf and root samples harvested on day 28 of the drought stress treatment. Three biological replicates were used for each tissue sample per treatment and wheat variety. Superoxide anion and H_2_O_2_ content analyses were conducted to assess the levels of oxidative stress-causing molecules in the drought-stressed plants, while the MDA content was used as a proxy for oxidative stress damage by lipid peroxidation.

Trichloroacetic acid extracts of each tissue sample were prepared for use in the MDA and H_2_O_2_ content analyses. Briefly, 100 mg of frozen leaf and root tissues were separately ground and homogenized in 5 mL of 6% (*w*/*v*) trichloroacetic acid (TCA), vortexed and centrifuged at 15,000× *g* for 10 min. The resultant supernatant was used as the TCA extract for the respective assays. MDA formation was assessed using the thiobarbituric acid (TBA) method as previously described [80]. The H_2_O_2_ content was estimated as described previously [80] with minor modifications. The reaction mixture containing 93.8 µL of the TCA extract, 187.5 µL 20 mM K_2_HPO_4_ (pH 5), and 375 µL 500 mM potassium iodide in a final volume of 750 µL was incubated for 20 min at 25 °C. Hydrogen peroxide standards were also prepared and processed as described previously [81]. Thereafter, absorbance readings of all samples were measured at 390 nm. The superoxide anion content was determined following a method described by Gokul et al. [80]. The supernatant was analyzed at 600 nm and superoxide content was calculated using the nitroblue tetrazolium (NBT) extinction coefficient of 12.8 mM^−1^cm^−1^.

#### 4.3.3. Proline and Glycine Betaine Content

Proline and glycine betaine content was determined for leaf and root samples using the hydrophilic interaction chromatography in tandem with liquid chromatography-mass spectrometry (HILIC LC-MS) as described previously [52,82]. The assays were performed on a QTRAP 6500 MS (Applied Biosystems Sciex, Foster City, CA, United States of America (USA)) following procedures described in our previous study [52]. Three biological replicates of tissue samples harvested on days 0, 14, and 28 of drought stress treatment were used. Leaf discs of 7 mm in diameter and 50 mg of ground frozen root samples were used for metabolite extraction and analyses.

#### 4.3.4. Enzymatic Antioxidant Activity Assays

Antioxidant enzymatic activity assays were determined for leaf and root samples harvested on day 28 of drought stress. All assays used three biological replicates of each tissue sample, per treatment and wheat variety. Plant tissues were separately ground to a fine powder using ice-cold mortar and pestles. The ground tissue of 100 mg was homogenized in 500 µL extraction buffer containing 40 mM phosphate buffer (pH 7.4), 1 mM ethylenediaminetetraacetic acid (EDTA), and 5% polyvinylpyrrolidone, vortexed and centrifuged at 9000× *g* for 5 min. The resultant supernatant was used as the enzyme extract, and protein concentrations were determined using a modified Bradford assay [83].

Superoxide dismutase (SOD) activity was determined as described previously [80]. Briefly, 10 µL of the enzyme extract was mixed with 300 µL 20 mM phosphate buffer (pH 7.8), 100 µL 0.1 mM EDTA, 100 µL 10 mM methionine, 100 µL 0.005 mM riboflavin, 100 µL 0.1 mM NBT, and 90 µL distilled water in a final volume of 800 µL. The reaction mixture was incubated at 25 °C for 20 min under light exposure and absorbance readings were taken at 560 nm. One unit of SOD activity was calculated based on the amount of enzyme required to cause a 50% reduction of NBT.

Ascorbate peroxidase (APX) activity was determined as described by Moloi and van der Merwe [84]. The reaction mixture contained 530 µL 50 mM potassium phosphate buffer (pH 7.0), 150 µL 0.5 mM ascorbate, 50 µL 0.1 mM EDTA, 200 µL 0.1 mM H_2_O_2_, 70 µL for leaf enzyme extract in a final volume of 1 mL. For the root samples, a 50 µL enzyme extract was used in a similar reaction mixture described above. The blank solution contained all reagents except for the enzyme extracts. Absorbance readings were measured at 290 nm, and the APX activity was calculated using an extinction coefficient of 2.8 mM^−1^cm^−1^.

Guaiacol peroxidase (GPX) activity was determined as described previously [84]. The reaction mixture of 1 mL consisted of 505 µL 80 mM phosphate buffer (pH 5.5), 100 µL 50 mM guaiacol, 50 µL 20 mM H_2_O_2_, 5 µL leaf enzyme extract, and 340 µL distilled water. For root samples, a 2 µL root enzyme extract was used in a 1 mL reaction mixture. The blank solution contained all reagents except for the enzyme extracts. The GPX activity was measured by observing an increase in absorbance at 470 nm over 3 min at 30 °C using a UV—visible spectrophotometer (Cary 100 Bio, Varian, Australia), using an extinction coefficient of 26.6 mM^−1^cm^−1^.

### 4.4. Leaf Protein Extraction, iTRAQ Labeling, LC-MS/MS, and Identification

#### 4.4.1. Leaf Protein Extraction, iTRAQ Labeling, and Tandem Mass Spectrometry

Proteomic analysis was conducted on leaf samples of both wheat varieties harvested on day 28 of the drought stress treatment. Four biological replicates were used for each treatment group per wheat variety. Total soluble proteins were extracted from 1 g of frozen leaf samples and quantified following previously described methods [83].

Full details of all protocols used for iTRAQ labeling, trypsin digestion, and protein identification by liquid-chromatography tandem mass spectrometry (LC-MS/MS) are described in our previous publication [52]. Briefly, 10 μg of each protein sample was prepared for labeling using an iTRAQ Reagent-Multiplex Buffer Kit (Applied Biosystems Sciex, Foster City, CA, USA) following the manufacturer’s protocol. Protein samples were subsequently digested with trypsin overnight at 37 °C and labeled with an 8-plex ITRAQ Reagent Kit (Applied Biosystems Sciex) following the manufacturer’s protocol with minor modifications [52]. Peptides of the four control samples of each wheat variety were separately labeled with iTRAQ tags 113, 114, 115, and 116, while those of the drought-stressed samples were labeled with tags 117, 118, 119, and 121. The four control and four drought-stressed labeled leaf samples of each wheat variety were pooled into one composite sample prior to sample clean-up and LC-MS/MS. Tandem mass spectrometry was performed on a TripleTOF 6600 spectrometer (Applied Biosystems Sciex) linked to an Eksigent 425 LC system via a Duospray source (Applied Biosystems Sciex, Foster City, CA, USA). Mass spectrometry data were acquired using the Applied Biosystems Sciex Analyst TF 1.7.1 instrument control and data processing software.

#### 4.4.2. Protein Identification and Quantification

Mass spectrometry protein identification was performed against the TrEMBL database for *T. aestivum* only with sequences downloaded in July 2022. The MS data analysis settings are as fully described by Goche et al. [52]. Peptide and protein tables were exported from the ProteinPilot 5.01 version 4895 software with the Paragon Algorithm 5.0.1.0.4874 and manually processed and filtered. In this study, all positively identified proteins were identified based on at least one peptide. This gave rise to 1062 and 882 positively identified proteins in the leaf extracts of BW4074 and BW35695, respectively. The relative expression of the drought-responsive leaf proteins is presented as fold change, calculated as a ratio to the 113-tagged control sample of each wheat variety. Upregulated proteins are given as positive values above 1, while downregulated proteins are denoted with negative fold change. The drought-responsive proteins were statistically analyzed using a Student’s *t*-test at *p* ≤ 0.05.

#### 4.4.3. Bioinformatics Analyses of Differentially Expressed Proteins

The drought-responsive leaf proteins were ascribed with Gene Ontology (GO) terms and protein family names using the UniProt [85] and InterPro [86] databases, respectively. The differentially expressed proteins were assigned functional groups using the classification scheme suggested by Bevan et al. [48]. We analyzed the functions of the drought-responsive proteins using a pathway enrichment analysis on the Kyoto Encyclopedia of Genes and Genomes (KEGG) database [87] using gene IDs as input data. Protein–protein interactions were predicted on the STRING (Search Tool for the retrieval of Interacting Genes/Proteins) version 12.0 database [88] using protein accessions as input data. The pathway enrichment analysis and protein–protein interactions were conducted using default settings of each bioinformatic tool. The UniProt and InterPro databases were accessed on 6 June 2024, while the KEGG and STRING databases were accessed on 20 June 2024.

### 4.5. Gene Expression Analysis

Total RNA was extracted from leaf samples of the control and drought-stressed plants of both wheat varieties following 28 days of stress treatment for use in gene expression analysis. Day 28 leaf samples were used for gene expression to correspond with the harvest time used for the proteome analysis. Three biological replicates were used for each treatment and wheat variety. Total RNA extraction with a DNase digestion step were performed using the Spectrum™ Plant Total RNA kit (Sigma Aldrich, St. Louis, MI, USA) and the On-column DNase 1 Digestion Set (Sigma), respectively. The GoScript Reverse Transcriptase System (Promega, Southampton, UK) was used for complementary DNA (cDNA) synthesis on a 1 μg total RNA template.

Quantitative real-time PCR (qRT-PCR) was performed using the SsoAdvanced Universal SYBR Green Supermix kit (Biorad, Hercules, CA, USA) following the manufacturer’s protocol. Reaction mixtures were prepared and run on a CFX Connect Real-Time System (Biorad) using the reaction component volumes and thermal cycling conditions described by Ngwenya et al. [89]. Two *T. aestivum* reference control genes, a wheat α-tubulin [90] and elongation factor-1 alpha [91], were used for data analysis performed on the CFX Maestro software version 4.1.2433.1219 (Biorad). Primer sequences (Table S1) were designed using the Primer-BLAST tool [92], synthesized and supplied by Inqaba Biotechnical Industries (Menlo Park, South Africa). Target genes for qRT-PCR analysis were randomly selected from the drought-responsive leaf proteins identified in the current study (Tables S6 and S7).

### 4.6. Statistical Analysis

A two-way analysis of variance (ANOVA) test was used to statistically analyze the data unless stated otherwise. In cases where three factors of stress type (control and drought stress), duration of stress treatment (across the different sampling times), and wheat genotypes (drought-susceptible and drought-tolerant) were analyzed, three-way ANOVA was performed. Means were compared according to the Tukey multiple comparison test at *p* ≤ 0.05. The ANOVA and Tukey multiple comparison statistical tests were performed using GraphPad Prism analysis software version 8.0.2.263.

## 5. Conclusions

We used two wheat varieties, the drought-tolerant BW35695 and the drought-sensitive BW4074, to gain insights into the physiological and molecular basis for drought tolerance in wheat. Our results suggest that when the plants were deprived of water for 28 days, the drought-tolerant variety maintains a higher leaf water content, possibly by accumulating higher levels of osmolytes such as proline, than the drought-sensitive variety. In addition, BW35695 protected its cell components from oxidative stress damage by limiting ROS accumulation and enhancing the enzymatic antioxidant capacity in plant tissues.

Our leaf proteomic results further suggest that drought stress modulates various cellular processes in both wheat varieties. Some of the identified drought-responsive proteins have putative functions in primary and secondary metabolism, protein synthesis/folding/degradation, defense/ROS detoxification, energy production, transcription, and cell structure. However, the number of the up- and downregulated proteins varied between functional groups within and between the two wheat varieties.

Nevertheless, we observed two noteworthy trends in the proteome data of the drought-tolerant variety BW35695: proteins involved in protein synthesis were highly upregulated, while those related to photosynthesis were downregulated. Although further functional validation of these proteomic results is required, it is plausible that the BW35695 responds to drought stress by reprogramming protein synthesis to generate proteins with regulatory and/or protective roles against the primary and secondary effects of drought. On the other hand, photosynthesis is pivotal in generating carbohydrates and energy for the growth and development of plants. However, during drought stress, photosynthesis may generate excess ROS, which exacerbates drought-induced oxidative stress and its adverse effects on plant cell structure and function. Therefore, the observed downregulation of proteins involved in photosynthesis in BW35695 might be a protective mechanism of this variety against oxidative stress in plants under drought. The effects of diminished photosynthetic capacity on yield losses of wheat plants under drought ought to be further investigated in field studies.

Apart from the future functional validation of these proteomic results using a broader range of enzyme assays and transgenic plant biology of a few target genes from these data, we propose more time-course gene expression analysis studies to investigate the transcriptional regulation in the two wheat varieties under drought conditions. In addition, future studies could analyze the drought responses of the root transcriptome, proteome and metabolome of these two wheat varieties to gain insights into the below-ground stress responses. Meta-analysis of datasets across different crops could also help to rank drought-adaptive proteins in different tissues and prioritize targets with predicted essential roles for reverse genetic analysis. Our results make an important contribution towards such an endeavor and the functional validation of candidate genes would ascertain the roles of selected proteins in plant stress response.

## Figures and Tables

**Table 1:** Summary list of wheat leaf proteome data obtained using iTRAQ and LC-MS/MS.

Wheat Variety	Positively Identified Proteins	Drought-Responsive Proteins	Protein Regulation	
			Up	Down
BW4074	1062	69	41	28
BW35695	882	110	58	52

**Table 2:** List of drought-responsive leaf proteins of the drought-susceptible wheat variety BW4074 with a minimum fold change of 1.5.

Accession ^a^	Protein Name ^b^	Ratio ^c^	SD ^d^	*p*-Value ^e^	Family Name ^f^
**Primary Metabolism**					
A0A3B6QKI8	GDSL esterase/lipase	−1.86	0.22	1.29 × 10^−2^	GDSL lipase/esterase-like, plant
A0A1D5URN5	Fibronectin type III-LIKE domain-containing protein	−1.55	0.08	2.99 × 10^−2^	Beta-D-xylosidase
A0A077RPJ4	Tryptophan synthase	−1.67	0.19	4.51 × 10^−2^	Tryptophan synthase, alpha chain
Q45NB2	Glutamine synthetase	−1.68	0.26	3.75 × 10^−2^	Glutamine synthetase
A0A3B6FGE7	Enoyl reductase (ER) domain-containing protein	1.89	0.41	2.32 × 10^−2^	Medium-chain dehydrogenase/ reductase
A0A3B6DHI0	Glutamate dehydrogenase	−2.08	0.19	4.52 × 10^−2^	Glutamate dehydrogenase
W5ACM8	Delta-1-pyrroline-5-carboxylate synthase	1.52	0.24	3.17 × 10^−2^	Delta-l-pyrroline-5-carboxylate synthetase
A0A3B6MSL2	Aminocyclopropanecarboxylate oxidase	1.58	0.36	2.09 × 10^−2^	Plant 2-oxoglutarate-dependent oxidoreductases
**Protein synthesis/folding/degradation**					
W5I1R7	30S ribosomal protein S3, chloroplastic	1.63	0.18	8.04 × 10^−3^	Small ribosomal subunit protein uS3
A0A3B6TJK6	Small ribosomal subunit protein uS10 domain-containing protein	1.64	0.40	2.86 × 10^−2^	Small ribosomal subunit protein uS10
W5H9B7	Peptidyl-prolyl cis-trans isomerase	−1.57	0.21	3.52 × 10^−2^	Cyclophilin-type peptidyl-prolyl cis-trans isomerase/CLD
A0A2 × 0SLI3	40S ribosomal protein S6	1.94	0.33	4.09 × 10^−2^	Small ribosomal subunit protein eS6-like
A0A3B6QHV4	Anion-transporting ATPase-like domain-containing protein	−1.68	0.25	2.63 × 10^−2^	Arsenical pump ATPase, ArsA/GET3
**Defense/ROS detoxification**					
A0A3B6HMK6	Glutathione reductase	1.94	0.66	4.69 × 10^−2^	Glutathione reductase
S6AWC2	Cold induced 16	1.68	0.42	4.43 × 10^−2^	Nodulin-related protein1/2
D8L9B5	Putative PDI-like protein	−2.57	0.11	1.11 × 10^−2^	Protein disulfide isomerase A6
**Energy**					
A0A3B5Z298	Phosphoglycerate kinase	−1.51	0.22	4.29 × 10^−2^	Phosphoglycerate kinase family
A0A3B6FUA0	Fructose-bisphosphate aldolase	1.71	0.27	2.64 × 10^−3^	Fructose-bisphosphate aldolase, class I
A0A3B6TBB9	Ferredoxin--NADP reductase, chloroplastic	−2.02	0.31	1.77 × 10^−2^	Ferredoxin-NADP reductase
A0A3B6KSW8	Glyceraldehyde-3-phosphate dehydrogenase	−1.68	0.18	4.84 × 10^−2^	Glyceraldehyde-3-phosphate dehydrogenase, type 1
A0A3B6BY66	Dihydrolipoyllysine-residue succinyltransferase	−1.61	0.18	3.67 × 10^−2^	Dihydrolipoamide succinyltransferase
A0A3B6BZB5	Transketolase	−1.88	0.04	1.89 × 10^−2^	Transketolase, bacterial-like
**Transcription**					
A0A3B6LV22	H15 domain-containing protein	1.50	0.19	4.14 × 10^−3^	Linker histone H1/H5
A0A3B6A3G3	Hyaluronan/mRNA-binding protein domain-containing protein	1.99	0.59	2.54 × 10^−2^	RNA binding protein HABP4/SERBP1-like
**Secondary metabolism**					
A0A3B6C785	Zeta-carotene desaturase	−1.50	0.18	4.20 × 10^−2^	Zeta-carotene desaturase
A0A3B6DPV0	AB hydrolase-1 domain-containing protein	−1.78	0.07	3.66 × 10^−2^	Epoxide hydrolase-like
**Cell structure**					
A0A3B6SEK0	Cyanobacterial aminoacyl-tRNA synthetase CAAD domain-containing protein	−1.87	0.19	2.29 × 10^−2^	Protein curvature thylakoid I
**Unclear classification**					
A0A3B6FKP0	PH domain-containing protein	1.71	0.34	7.96 × 10^−3^	Ricin B-like lectin EULS3-like
A0A3B5XZW3	Remorin C-terminal domain-containing protein	−1.65	0.02	1.98 × 10^−2^	None predicted

^a^ Protein accessions obtained from UniProt (https://www.uniprot.org/) database searches against sequences of *Triticum aestivum* only, accessed on 6 June 2024. ^b^ Protein names retrieved from the UniProt database on 6 June 2024. ^c^ Fold change in the stress-responsive proteins calculated relative to the control samples, each with four biological replicates. A positive value indicates upregulation, while a negative value indicates downregulation of the respective protein. ^d^ Standard deviation of the fold changes (*n* = 4). ^e^ Probability value (*p ≤* 0.05) obtained from a Student’s *t*-test comparing the fold changes between the drought stress treatment and the control (*n* = 4). ^f^ Protein family names retrieved from the InterPro (https://www.ebi.ac.uk/interpro/) database, accessed on 6 June 2024.

**Table 3:** List of drought-responsive leaf proteins of the drought-tolerant wheat variety BW35695 with a minimum fold change of 1.5.

Accession ^a^	Protein Name ^b^	Ratio ^c^	SD ^d^	*p*-Value ^e^	Family Name ^f^
**Primary Metabolism**					
A0A3B6UC94	Acid phosphatase	2.05	0.28	5.05 × 10^−4^	Acid phosphatase, plant
A0A3B6FKL0	Nucleoside phosphorylase domain-containing protein	1.66	0.27	3.78 × 10^−3^	Phosphorylase superfamily
A0A3B6B442	Aspartate/glutamate/uridylate kinase domain-containing protein	−1.92	0.34	4.08 × 10^−2^	Glutamate/acetylglutamate kinase
A0A3B6TUD9	Thiamine thiazole synthase, chloroplastic	−1.74	0.13	3.42 × 10^−2^	Thiamine thiazole synthase
A0A3B6NHD8	O-methyltransferase ZRP4	1.56	0.41	3.89 × 10^−2^	O-methyltransferase COMT-type
A0A3B5ZXG4	Glycosyltransferase	−1.50	0.12	2.23 × 10^−2^	UDP-glucuronosyl/UDP-glucosyltransferase
A0A3B6MS26	Glucose-6-phosphate 1-epimerase	−2.37	0.15	1.86 × 10^−3^	Glucose-6-phosphate 1-epimerase
A0A3B6KPK9	Beta-glucosidase	2.27	0.88	4.53 × 10^−2^	Cellulase degradation glycosyl hydrolase 3
**Energy**					
P24065	Photosystem II CP47 reaction center protein	−2.02	0.12	1.01 × 10^−2^	Photosystem II CP47 reaction centre protein
A0A3B6AYY2	23 kDa subunit of oxygen evolving system of photosystem II	1.56	0.35	4.27 × 10^−2^	PsbP
A0A3B6N1I7	Photosystem I reaction center subunit II, chloroplastic	−1.57	0.19	1.60 × 10^−2^	Photosystem I PsaD
A0A3B6LQN1	Chlorophyll A-B binding protein, chloroplastic	−1.63	0.11	2.36 × 10^−2^	Chlorophyll A-B binding protein
A0A3B6JMT4	Glyceraldehyde-3-phosphate dehydrogenase	−2.20	0.42	4.92 × 10^−2^	Glyceraldehyde-3-phosphate dehydrogenase, type 1
W5C4P1	Uncharacterized protein	−1.92	0.08	1.06 × 10^−2^	Oxygen-evolving enhancer protein 3, plants
W5AY52	Chlorophyll A-B binding protein, chloroplastic	−1.67	0.08	1.53 × 10^−3^	Chlorophyll A-B binding protein
W5D4R0	Photosystem II 22 kDa protein, chloroplastic	−1.63	0.13	5.52 × 10^−3^	Chlorophyll A-B binding protein
A0A3B6QKY1	Aconitate hydratase	2.05	0.47	5.72 × 10^−3^	Aconitase/Iron-responsive element-binding protein 2
P58386	Photosystem I P700 chlorophyll A apoprotein A2	−2.39	0.09	2.93 × 10^−3^	Photosystem I PsaB
A0A3B6QDB2	Photosystem II protein D1	−1.57	0.14	2.89 × 10^−2^	Photosynthetic reaction centre, L/M
P58311	Photosystem I P700 chlorophyll A apoprotein A1	−2.39	0.04	9.95 × 10^−3^	Photosystem I PsaA
A0A3B6HUR4	Chlorophyll A-B binding protein, chloroplastic	−1.91	0.09	4.14 × 10^−3^	Chlorophyll A-B binding protein
A0A3B6AWZ1	Chlorophyll A-B binding protein, chloroplastic	−1.58	0.05	3.46 × 10^−3^	Chlorophyll A-B binding protein
W5F8Z5	Chlorophyll A-B binding protein, chloroplastic	−1.69	0.07	1.88 × 10^−4^	Chlorophyll A-B binding protein
A0A3B5Z4J5	ATP synthase subunit b, chloroplastic OS = *Triticum aestivum*	−1.55	0.09	2.09 × 10^−2^	ATPase, FO complex, subunit b/b’
P69415	Photosystem I iron-sulfur center	−1.65	0.06	3.97 × 10^−2^	Photosystem I protein PsaC
A0A3B6PUD8	Photosystem II 10 kDa polypeptide, chloroplastic	−1.64	0.08	5.55 × 10^−3^	Photosystem II PsbR
F6K744	Chlorophyll A-B binding protein, chloroplastic	−2.27	0.06	1.71 × 10^−3^	Chlorophyll A-B binding protein
A0A3B6U9Q7	Cytochrome b/b6 C-terminal region profile domain-containing protein	−3.44	0.21	1.90 × 10^−2^	Cytochrome b6/f complex, subunit IV
**Protein synthesis/folding/degradation**					
W5ASA4	Uncharacterized protein	1.61	0.41	4.51 × 10^−2^	Small ribosomal subunit protein uS19
A0A3B6JIR3	Heat shock cognate 70kDa protein	1.55	0.34	4.59 × 10^−2^	Heat shock protein 70 family
A0A3B6B204	Large ribosomal subunit protein uL23 N-terminal domain-containing protein	1.59	0.16	5.55 × 10^−4^	Large ribosomal subunit protein uL23
W5D739	KOW domain-containing protein	1.64	0.24	4.49 × 10^−3^	Large ribosomal subunit protein uL24
A0A3B6MTE3	Peptidylprolyl isomerase	1.64	0.38	3.20 × 10^−2^	Peptidyl-prolyl cis-trans isomerase FKBP18-like
A0A3B6N0K3	50S ribosomal protein L17, chloroplastic	1.51	0.21	3.22 × 10^−3^	Large ribosomal subunit protein bL17
A0A3B6A2B9	60S ribosomal protein L37a	2.54	0.42	9.27 × 10^−3^	Large ribosomal subunit protein eL43
A0A3B6QGX5	60S ribosomal protein L6	1.65	0.29	4.00 × 10^−2^	Large ribosomal subunit protein eL6
A0A3B6UD00	50S ribosomal protein L20	1.83	0.45	1.28 × 10^−2^	Large ribosomal subunit protein bL20
**Transporters**					
A0A3B6GKQ2	Non-specific lipid-transfer protein	1.71	0.40	1.31 × 10^−2^	Plant non-specific lipid-transfer protein/Par allergen
A0A3B5YX09	Chloroplast inner envelope protein	−1.50	0.10	2.53 × 10^−2^	Protein TIC110, chloroplastic
A0A3B6I0D3	STI1/HOP DP domain-containing protein	1.74	0.39	1.84 × 10^−2^	None predicted
**Transcription**					
A0A3B6LSN3	MBD domain-containing protein	1.52	0.18	7.18 × 10^−3^	Methyl-CpG-binding domain-containing protein 10/11
Q8LRU5	HMG-I/Y protein HMGa	1.60	0.40	3.88 × 10^−2^	High-mobility group protein HMGA
A0A3B6MXZ6	H15 domain-containing protein	1.84	0.44	4.79 × 10^−2^	Linker histone H1/H5
A0A3B6GNG0	Histone H2B	1.53	0.24	5.14 × 10^−3^	Histone H2B
**Defense/ROS detoxification**					
A0A3B6EFA0	Uncharacterized protein	2.66	0.73	4.24 × 10^−3^	Nodulin-related protein ½ family
Q8W428	Chitinase	−1.56	0.17	9.40 × 10^−3^	Glycoside hydrolase, family 19
A0A172WCB1	Cold-responsive LEA/RAB-related COR protein	3.23	1.19	1.01 × 10^−2^	None predicted
A0A3B6TZ07	GH18 domain-containing protein	1.84	0.28	1.98 × 10^−3^	Glycoside hydrolase 18 family chitinases
A0A3B6MJX1	Pathogen-related protein	−2.00	0.13	1.68 × 10^−2^	Pathogen-related defense protein
**Secondary metabolism**					
A0A3B6TV37	Amine oxidase domain-containing protein	1.67	0.33	2.10 × 10^−2^	Flavin monoamine oxidase and related enzymes
A0A3B6QDX1	Delta-aminolevulinic acid dehydratase	−1.59	0.17	8.35 × 10^−3^	Delta-aminolevulinic acid dehydratase family
A0A3B5Y2F9	Dienelactone hydrolase domain-containing protein	1.60	0.22	2.30 × 10^−3^	Dienelactone hydrolase family
**Cell structure**					
W5FAY5	Actin	−1.50	0.15	3.53 × 10^−2^	Actin family
**Unclear classification**					
A0A3B6GTL4	DJ-1/PfpI domain-containing protein	−1.79	0.11	5.38 × 10^−4^	Protein/nucleic acid deglycase DJ-1
A0A3B6TRL4	Thylakoid membrane protein slr0575	−1.78	0.19	1.58 × 10^−2^	Protein of unknown function DUF2854
A0A3B6AVR1	Uncharacterized protein	−1.63	0,06	4.05 × 10^−2^	RidA family
A0A3B6N353	Pentacotripeptide-repeat region of PRORP domain-containing protein	2.10	0.41	3.42 × 10^−3^	Tetratricopeptide-like helical domain superfamily
W5CRR3	DUF538 domain-containing protein	−2.38	0.13	1.91 × 10^−3^	Protein of unknown function DUF538
A0A3B5ZXF0	Protein kinase domain-containing protein	1.92	0.44	9.42 × 10^−3^	None predicted

^a^ Protein accessions obtained from UniProt (https://www.uniprot.org/) database against sequences of *Triticum aestivum* only, accessed on 6 June 2024. ^b^ Protein names retrieved from the UniProt database on 6 June 2024. ^c^ Fold change in the stress-responsive proteins calculated relative to the control samples, each with four biological replicates. A positive value indicates upregulation, while a negative value indicates downregulation of the respective protein. ^d^ Standard deviation of the fold changes (*n* = 4). ^e^ Probability value (*p ≤* 0.05) obtained from a Student’s *t*-test comparing the fold changes between the drought stress treatment and the control (*n* = 4). ^f^ Protein family names retrieved from the InterPro (https://www.ebi.ac.uk/interpro/) database, accessed on 6 June 2024.

**Table 4:** List of drought stress-responsive proteins common to both BW4074 and BW35695 wheat varieties.

Accession	Protein Name	Ratio ^a^		BW4074 vs. BW35698 Ratio *p*-Value ^b^
		BW4074	BW35698	
**Primary Metabolism**				
A0A3B6MJZ2	5-methyltetrahydropteroyltriglutamate-homocysteine S-methyltransferase	1.47	1.37	3.39 × 10^−1^
A0A1D5URN5	Fibronectin type III-like domain-containing protein	−1.55	−1.15	3.30 × 10^−3^ *
**Protein synthesis/folding/degradation**				
A0A3B6TJK6	Small ribosomal subunit protein uS10 domain-containing protein	1.64	1.44	3.98 × 10^−1^
A0A3B6MTE3	Peptidylprolyl isomerase	1.28	1.64	1.28 × 10^−1^
W5D1D3	30S ribosomal protein S20, chloroplastic	−1.27	1.36	2.49 × 10^−3^ *
**Energy**				
A0A3B6QKY1	Aconitate hydratase	1.26	2.05	1.83 × 10^−2^ *
P69415	Photosystem I iron-sulfur center	−1.46	−1.65	4.52 × 10^−1^
**Transcription**				
A0A3B6LSN3	MBD domain-containing protein	1.36	1.52	2.86 × 10^−1^
**Unclear classification**				
A0A3B6MQA1	RRM domain-containing protein	1.20	−1.24	9.37 × 10^−4^ *

^a^ Fold change in each common protein as illustrated in Tables S6 and S7 for BW4074 and BW35695 wheat variety, respectively. ^b^ The *p*-value from a Student *t*-test (*p ≤* 0.05) comparing the fold change in each protein for the two wheat varieties. These data were extracted from Tables S6 and S7 for BW4074 and BW35695, respectively. * Proteins with significant differences in fold changes between the two wheat varieties according to a Student *t*-test (*p ≤* 0.05). The four biological replicate fold changes used in this analysis are those used to generate data shown in Tables S4 and S5 for BW4074 and BW35695, respectively.

## Data Availability

The datasets generated and/or analyzed during this study are available from the corresponding author on request.

## Supplementary Materials

The following supporting information can be downloaded at: https://www.mdpi.com/article/10.3390/plants13192797/s1, Figure S1: Effect of drought stress on the growth of wheat plants. (a) Shoot fresh weight; (b) shoot dry weight; (c) root fresh weight; (d) root dry weight; (e) shoot length; (f) root length. Two-week old plants of BW4074 (drought-susceptible) and BW35695 (drought-tolerant) wheat varieties were exposed to drought stress by withholding water for 28 days for the respective measurements. Data presented as mean ± SE (*n* = 3). Different letters indicate significant differences between means at (*p* ≤ 0.05) according to ANOVA and Tukey–Kramer test; Figure S2: Putative biological processes of the drought stress-responsive leaf proteins of wheat; Figure S3: Putative molecular functions of the drought stress-responsive leaf proteins of wheat; Table S1: List of leaf wheat primer sequences used in gene expression analysis; Table S2: List of leaf proteins identified in BW4074 wheat variety; Table S3: List of leaf proteins identified in BW35695 wheat variety; Table S4: List of differentially expressed leaf proteins in BW4074 in response to drought stress; Table S5: List of differentially expressed leaf proteins in BW35695 in response to drought stress; Table S6: List of drought-responsive leaf proteins of the drought-susceptible wheat variety BW4074 identified using iTRAQ and LC-MS/MS; Table S7: List of drought-responsive leaf proteins of the drought-tolerant wheat variety BW35695 identified using iTRAQ and LC-MS/MS.
