# Supplementary material for: Comparative Physiological, Biochemical, and Leaf Proteome Responses of Contrasting Wheat Varieties to Drought Stress
Source: Plants (Basel). 2024 Oct 5;13(19):2797. doi: 10.3390/plants13192797 (PMC11478804; doi:10.3390/plants13192797)
Supplement: Supplementary file 1 [file plants-13-02797-s001.zip › Table S1.pdf]

Table S1: List of leaf wheat primer sequences used in gene expression analysis.

*Accession No.	Protein name	Forward Primer (5'>3')	Reverse Primer (5'>3')
A0A3B6U9Q7	CYTB_CTER domain-containing protein	AGTTGTAGGTGCAACTTCGG	GGCTCGAGCAAGAGTGAAAG
A0A3B6N353	Pentacotripeptide-repeat region of PRORP domain-containing protein	CGGCATGAGGCTGTTTTCTG	CCACAGAGGTGTAGTGCTCC
A0A3B6EFA0	Uncharacterized protein	AGCTGGTGATATCTGGTTCTG	ACAACCGAAATCCTGCAATCG
P58311	Photosystem I P700 chlorophyll a apoprotein A1	GAGTGCTTTAGGCCGTCCAC	TCGCAAGGAAAGCGAAAACC
W5ACM8	Delta-1-pyrroline-5-carboxylate synthase	CTCGGCCAGGAGATAAACTCG	AGCACGTCCTACAGGTTTGG
A0A3B6TZ07	GH18 domain-containing protein	CAGATTGGTCGCACAGTTATGG	TCTTGCATTTGCTGCTGTGC
Refence gene	Wheat α-tubulin	ATCTGTGCCTTGACCGTATCAGG	GACATCAACATTCAGGACACCATC
Reference gene	Elongation factor 1-α	GCTGTCAAGTTTGCTGAGATCC	GTACTGAGCGAAGGTCTCCAC

*Accession No. obtained from iTRAQ proteomic data.
