# Supplementary material for: Comparative Physiological, Biochemical, and Leaf Proteome Responses of Contrasting Wheat Varieties to Drought Stress
Source: Plants (Basel). 2024 Oct 5;13(19):2797. doi: 10.3390/plants13192797 (PMC11478804; doi:10.3390/plants13192797)
Supplement: Supplementary file 1 [file plants-13-02797-s001.zip › Tables S6.pdf]

Table S6: List of drought-responsive leaf proteins of the drought susceptible wheat variety BW4074 identified using iTRAQ and LC-MS/MS.

Accession[a]	Protein name[b]	Scor[c]	% Cov[d]	Seq. Pep[e]	Ratio[f]	SD[g]	p-value[h]	Gene ontology analysis[i]			Family name[j]
								P	F	C	
**Primary metabolism**											
A0A3B6MJZ2	5-methyltetrahydropteroyltriglutamate-homocysteine S-methyltransferase OS=*Triticum aestivum*	38.07	30.26	20	1.47	0.07	1.59×10^{-4}	Methionine biosynthetic process	Methyltransferase activity	None	Cobalamin-independent methionine synthase
A0A3B6DM57	Lipoxygenase OS=*Triticum aestivum*	46.66	24.49	25	1.22	0.09	5.04×10^{-3}	Fatty acid biosynthetic process	Oxidoreductase activity	None	Lipoxygenase, plant
A0A3B6GPF7	Glutamate decarboxylase OS=*Triticum aestivum*	12.57	18.16	8	1.29	0.12	6.48×10^{-3}	Glutamate metabolic process	Glutamate decarboxylase activity	Cytosol	Glutamate decarboxylase
A0A3B6JRZ2	Apyrase OS=*Triticum aestivum*	10.46	12.1	5	1.36	0.29	4.90×10^{-2}	Nucleoside diphosphate catabolic process	Nucleoside diphosphate phosphatase activity	Membrane	Nucleoside phosphatase GDA1/CD39
A0A3B6U5R3	Acid phosphatase OS=*Triticum aestivum*	13.55	25.71	8	1.44	0.18	1.87×10^{-2}	None	Acid phosphatase activity	None	Acid phosphatase, plant
A0A3B6C6N3	Beta-amylase OS=*Triticum aestivum*	8.08	11.66	4	-1.27	0.07	1.31×10^{-2}	Polysaccharide catabolic process	Beta-amylase activity	None	Glycosyl hydrolase, family 14B plant
A0A3B6QIZ3	S-adenosylmethionine synthase OS=*Triticum aestivum*	7.28	9.898	5	1.35	0.09	2.99×10^{-3}	S-adenosylmethionine biosynthetic process	Methionine adenosyltransferase activity	Cytosol	S-adenosyl methionine synthetase
A0A3B6H4R5	2-hydroxyacyl-CoA lyase OS=*Triticum aestivum*	6.44	8.377	3	1.39	0.11	1.36×10^{-2}	Fatty acid alpha-oxidation	Thiamine pyrophosphate binding	Peroxisome	TPP-binding domain containing protein HACL1-like
A0A3B6QKI8	GDSL esterase/lipase OS=*Triticum aestivum*	4	13.67	2	-1.86	0.22	1.29×10^{-2}	None	Hydrolase activity, acting on ester bonds	None	GDSL lipase/esterase-like, plant
A0A1D5URN5	Fibronectin type III-LIKE domain-containing protein OS=*Triticum aestivum*	3.97	4.534	2	-1.55	0.08	2.99×10^{-2}	Xylan catabolic process	Xylan 1,4-beta-xylosidase activity	Extracellular region	Beta-D-xylosidase
A0A077RPJ4	Tryptophan synthase OS=*Triticum aestivum*	2.19	10.85	2	-1.67	0.19	4.51×10^{-2}	Tryptophan biosynthetic process	Tryptophan synthase activity	Cytosol	Tryptophan synthase, alpha chain
Q45NB2	Glutamine synthetase OS=*Triticum aestivum*	17.88	39.11	15	-1.68	0.26	3.75×10^{-2}	Glutamate biosynthetic process	Glutamine synthetase activity	Cytoplasm	Glutamine synthetase

A0A3B6FGE7	Enoyl reductase (ER) domain-containing protein OS=*Triticum aestivum*	2	7.062	1	1.89	0.41	2.32×10^{-2}	None	Oxidoreductase activity	None	Medium-chain dehydrogenase/ reductase
A0A3B6DHI0	Glutamate dehydrogenase OS=*Triticum aestivum*	2	2.691	1	-2.08	0.19	4.52×10^{-2}	Glutamate catabolic process	Glutamate dehydrogenase activity	Mitochondrion	Glutamate dehydrogenase
W5ACM8	Delta-1-pyrroline-5-carboxylate synthase OS=*Triticum aestivum*	1.73	1.676	1	1.52	0.24	3.17×10^{-2}	proline biosynthetic process	Glutamate-5-semialdehyde dehydrogenase activity	Cytoplasm	Delta-l-pyrroline-5-carboxylate synthetase
A0A3B6MSL2	Aminocyclopropanecarboxylate oxidase OS=*Triticum aestivum*	1.1	4.389	1	1.58	0.36	2.09×10^{-2}	None	Oxidoreductase activity	None	Plant 2-oxoglutarate-dependent oxidoreductases
**Protein synthesis/folding/degradation**											
W5CXB0	Heat shock 70 kDa protein, mitochondrial OS=*Triticum aestivum*	27.46	22.81	14	1.12	0.04	4.01×10^{-2}	Protein refolding	Unfolded protein binding	Cytoplasm	Heat shock protein 70 family
Q5I7L1	Ribosomal protein L13a OS=*Triticum aestivum*	9.56	22.82	5	1.18	0.06	1.57×10^{-2}	Translation	Structural constituent of ribosome	Ribosome	Large ribosomal subunit protein uL13, eukaryotic/archaeal
Q5I7K4	Ribosomal protein L17 OS=*Triticum aestivum*	7.12	25	4	1.19	0.10	1.99×10^{-2}	Translation	Structural constituent of ribosome	Ribosome	Large ribosomal subunit protein uL14
E2F3W4	Small ribosomal subunit protein uS8c OS=*Triticum aestivum*	6.89	23.08	4	-1.19	0.04	3.21×10^{-3}	Translation	Structural constituent of ribosome	Ribosome	Small ribosomal subunit protein uS8
A0A3B6MTE3	Peptidylprolyl isomerase OS=*Triticum aestivum*	6.3	18.92	3	1.28	0.15	3.72×10^{-2}	None	Peptidyl-prolyl cis-trans isomerase activity	None	Peptidyl-prolyl cis-trans isomerase FKBP18-like
W5I1R7	30S ribosomal protein S3, chloroplastic OS=*Triticum aestivum*	4.06	13.22	2	1.63	0.18	8.04×10^{-3}	Translation	Structural constituent of ribosome	Ribosome	Small ribosomal subunit protein uS3
A0A3B6TJK6	Small ribosomal subunit protein uS10 domain-containing protein OS=*Triticum aestivum*	3.54	12.26	2	1.64	0.40	2.86×10^{-2}	Translation	Structural constituent of ribosome	Ribosome	Small ribosomal subunit protein uS10
A0A3B6A2M8	50S ribosomal protein L12, chloroplastic OS=*Triticum aestivum*	3.49	13.97	2	-1.38	0.12	2.42×10^{-2}	Translation	Structural constituent of ribosome	Ribosome	Large ribosomal subunit protein bL12
W5H9B7	Peptidyl-prolyl cis-trans isomerase OS=*Triticum aestivum*	3	13.3	2	-1.57	0.21	3.52×10^{-2}	Protein folding	Peptidyl-prolyl cis-trans isomerase activity	Cytoplasm	Cyclophilin-type peptidyl-prolyl cis-trans isomerase/CLD

A0A2X0SLI3	40S ribosomal protein S6 OS=*Triticum aestivum*	4.65	11.6	2	1.94	0.33	4.09×10^{-2}	Translation	Structural constituent of ribosome	Ribosome	Small ribosomal subunit protein eS6-like
W5D1B3	Trigger factor ribosome-binding bacterial domain-containing protein OS=*Triticum aestivum*	1.77	4.4	1	-1.47	0.20	4.54×10^{-2}	Protein folding	Peptidyl-prolyl cis-trans isomerase activity	None	Trigger factor
W5D1D3	30S ribosomal protein S20, chloroplastic OS=*Triticum aestivum*	2.17	3.191	1	-1.27	0.08	2.50×10^{-2}	Translation	Structural constituent of ribosome	Ribosome	Small ribosomal subunit protein bS20
A0A3B6QHV4	Anion-transporting ATPase-like domain-containing protein OS=*Triticum aestivum*	0.84	3.275	1	-1.68	0.25	2.63×10^{-2}	Post-translational protein targeting to ER membrane	ATP hydrolysis activity	GET complex	Arsenical pump ATPase, ArsA/GET3
A0A3B6HY00	Sigma 54 modulation/S30EA ribosomal protein C-terminal domain-containing protein OS=*Triticum aestivum*	7.26	20.13	6	1.29	0.20	4.50×10^{-2}	Negative regulation of translation elongation	Ribosomal small subunit binding	Cytosolic small ribosomal subunit	Ribosome hibernation promoting factor, long/plastid
**Defence/ROS detoxification**											
Q96123	Superoxide dismutase [Cu-Zn] OS=*Triticum aestivum*	10.63	46.77	10	1.32	0.07	1.06×10^{-3}	Removal of superoxide radicals	Superoxide dismutase activity	Chloroplast	Superoxide dismutase (Cu/Zn) / superoxide dismutase copper chaperone
A0A3B6HMK6	Glutathione reductase OS=*Triticum aestivum*	10.49	17.45	7	1.94	0.66	4.69×10^{-2}	Cell redox homeostasis	Glutathione-bisulfide (NADPH) reductase activity	Cytoplasm	Glutathione reductase
A7VL25	Group3 late embryogenesis abundant protein OS=*Triticum aestivum*	5.9	18.08	3	1.38	0.07	3.02×10^{-2}	None	None	None	None predicted
O82715	Pathogenesis related protein-1.2 OS=*Triticum aestivum*	4.04	21.97	4	-1.29	0.02	2.53×10^{-3}	Response to biotic stimulus	None	Extracellular space	Venom allergen 5-like
A0A3B6CH98	Peroxidase OS=*Triticum aestivum*	4	7.038	2	-1.48	0.10	2.23×10^{-2}	Response to oxidative stress	Peroxidase activity	Extracellular region	Plant peroxidase
S6AWC2	Cold induced 16 OS=*Triticum aestivum*	4.06	13.29	2	1.68	0.42	4.43×10^{-2}	Regulation of abscisic acid biosynthetic process	None	None	Nodulin-related protein1/2
H9NAV6	Superoxide dismutase [Cu-Zn] OS=*Triticum aestivum*	2.95	13.16	1	1.45	0.34	4.82×10^{-2}	Removal of superoxide radicals	Superoxide dismutase activity	None	Superoxide dismutase (Cu/Zn) / superoxide dismutase copper chaperone

A0A3B6TKC6	Monodehydroascorbate reductase (NADH) OS=*Triticum aestivum*	2.16	5.357	2	1.32	0.16	3.66×10^{-2}	None	Oxidoreductase activity	Cytoplasm	FAD-dependent oxidoreductases and apoptosis regulators
D8L9B5	Putative PDI-like protein OS=*Triticum aestivum*	1.46	2.955	1	-2.57	0.11	1.11×10^{-2}	Response to endoplasmic reticulum stress	Protein-disulfide reductase activity	Endoplasmic reticulum lumen	Protein disulfide isomerase A6
**Energy**											
A0A3B5Z298	Phosphoglycerate kinase OS=*Triticum aestivum*	49.11	39.26	30	-1.51	0.22	4.29×10^{-2}	Glycolytic process	Phosphoglycerate kinase activity	Cytosol	Phosphoglycerate kinase family
A0A3B6FUA0	Fructose-bisphosphate aldolase OS=*Triticum aestivum*	20.09	35.57	14	1.71	0.27	2.64×10^{-3}	Glycolytic process	Fructose-bisphosphate aldolase activity	Cytosol	Fructose-bisphosphate aldolase, class I
A0A3B6TBB9	Ferredoxin--NADP reductase, chloroplastic OS=*Triticum aestivum*	15.94	30.11	13	-2.02	0.31	1.77×10^{-2}	Photosynthesis	Ferredoxin-NADP+ reductase activity	Chloroplast	Ferredoxin-NADP reductase
A0A3B6KSW8	Glyceraldehyde-3-phosphate dehydrogenase OS=*Triticum aestivum*	23.65	32.21	22	-1.68	0.18	4.84×10^{-2}	Glucose metabolic process	Glyceraldehyde-3-phosphate dehydrogenase activity	None	Glyceraldehyde-3-phosphate dehydrogenase, type 1
A0A3B6QKY1	Aconitate hydratase OS=*Triticum aestivum*	14.01	9.026	7	1.26	0.16	4.17×10^{-2}	Citrate metabolic process	Aconitate hydratase activity	Cytosol	Aconitase/Iron-responsive element-binding protein 2
A0A3B6QBJ3	Acetyltransferase component of pyruvate dehydrogenase complex OS=*Triticum aestivum*	9.67	7.38	6	-1.42	0.15	2.45×10^{-2}	Acetyl-CoA biosynthetic process from pyruvate	Acetyltransferase activity	Cytoplasm	Dihydrolipollysine-residue acetyltransferase component of pyruvate dehydrogenase complex
A0A3B6LW58	Glucose-1-phosphate adenylyltransferase OS=*Triticum aestivum*	9.36	12.55	5	1.21	0.09	3.34×10^{-2}	Starch biosynthetic process	Glucose-1-phosphate adenylytransferase activity	Chloroplast	Glucose-1-phosphate adenylytransferase
A0A3B6TJ01	Pyruvate dehydrogenase E1 component subunit beta OS=*Triticum aestivum*	8.87	16.12	5	1.43	0.16	4.63×10^{-2}	Acetyl-CoA biosynthetic process from pyruvate	Pyruvate dehydrogenase activity	Pyruvate dehydrogenase complex	Pyruvate dehydrogenase E1 component subunit beta
A0A3B6RKE8	Glyceraldehyde-3-phosphate dehydrogenase OS=*Triticum aestivum*	20.62	34.2	17	1.33	0.20	3.54×10^{-2}	Glucose metabolic process	Glyceraldehyde-3-phosphate dehydrogenase activity	Cytosol	Glyceraldehyde-3-phosphate dehydrogenase, type 1
A0A3B6QKZ9	ATP synthase delta chain, chloroplastic OS=*Triticum aestivum*	6.34	32.4	6	-1.27	0.08	8.97×10^{-3}	ATP biosynthetic process	Proton-transporting ATP synthase	Membrane	ATPase OSCP/delta subunit

									activity, rotational mechanism		
A0A3B6MR18	Phosphoenolpyruvate carboxylase OS=*Triticum aestivum*	4.8	2.366	2	1.37	0.23	4.38×10^{-2}	Tricarboxylic acid cycle	Phosphoenolpyruvate carboxylase activity	Cytosol	PEPCase type 1
P69415	Photosystem I iron-sulfur center OS=*Triticum aestivum*	2.86	19.75	3	-1.46	0.19	3.80×10^{-2}	Photosynthesis	4 iron, 4 sulfur cluster binding	Photosystem I	Photosystem I protein PsaC
A0A3B6BY66	Dihydrolipoyllysine-residue succinyltransferase OS=*Triticum aestivum*	2.04	4.684	1	-1.61	0.18	3.67×10^{-2}	Tricarboxylic acid cycle	Dihydrolipoyllysine-residue succinlytransferase activity	Mitochondrion	Dihydrolipoamide succinyltransferase
Q35980	30 kDa subunit of complex I NADH dehydrogenase OS=*Triticum aestivum*	2	3.833	1	-1.24	0.05	1.10×10^{-2}	Electron transport chain	NADH dehydrogenase (ubiquinone) activity	Mitochondrion	None predicted
A0A3B6EQ45	Uncharacterised protein OS=*Triticum aestivum*	0.74	3.712	2	1.28	0.12	2.73×10^{-2}	Pentose-phosphate shunt	Transaldolase activity	Cytoplasm	Transaldolase type 2
A0A3B6BZB5	Transketolase OS=*Triticum aestivum*	4.85	1.42	2	-1.88	0.04	1.89×10^{-2}	Pentose-phosphate shunt	Transketolase activity	Cytosol	Transketolase, bacterial-like
**Transcription**											
A0A3B6LSN3	MBD domain-containing protein OS=*Triticum aestivum*	3.44	5.684	2	1.36	0.20	3.57×10^{-2}	Transcription regulation	DNA binding	Nucleus	Methyl-CpG binding domain-containing protein 10/11
A0A3B6LV22	H15 domain-containing protein OS=*Triticum aestivum*	11.01	18.97	6	1.50	0.19	4.14×10^{-3}	Negative regulation of DNA recombination	Nucleosomal DNAbinding	Nucleus	Linker histone H1/H5
A0A3B6A3G3	Hyaluronan/mRNA-binding protein domain-containing protein OS=*Triticum aestivum*	0.84	4.011	1	1.99	0.59	2.54×10^{-2}	None	RNA binding	Nucleus	RNA binding protein HABP4/SERBP1-like
**Secondary metabolism**											
A0A3B6QF91	Phenylalanine ammonia-lyase OS=*Triticum aestivum*	9.34	10.65	5	1.39	0.09	5.53×10^{-3}	L-phenylalanine catabolic process	Phenylalanine ammonia-lyase activity	Cytoplasm	Phenylalanine ammonia-lyase
A0A3B6IV90	CN hydrolase domain-containing protein OS=*Triticum aestivum*	8.75	16.38	4	1.10	0.04	1.26×10^{-2}	Oxaloacetate metabolic process	Omega-amidase activity	None	Carbon-nitrogen hydrolase superfamily
A0A3B6C785	Zeta-carotene desaturase OS=*Triticum aestivum*	2.93	4.401	2	-1.50	0.18	4.20×10^{-2}	Carotenoid biosynthetic process	Oxidoreductase activity	Chloroplast	Zeta-carotene desaturase

A0A3B6DPV0	AB hydrolase-1 domain-containing protein OS=*Triticum aestivum*	2	5.764	1	-1.78	0.07	3.66×10^{-2}	None	Catalytic activity	None	Epoxide hydrolase-like
**Cell structure**											
A0A3B6SEK0	Cyanobacterial aminoacyl-tRNA synthetase CAAD domain-containing protein OS=*Triticum aestivum*	2.05	10.76	2	-1.87	0.19	2.29×10^{-2}	None	None	Chloroplast thylakoid membrane	Protein curvature thylakoid I
**Unclear classification**											
A0A3B5XTY5	Cupin type-1 domain-containing protein OS=*Triticum aestivum*	12.77	21.51	7	1.35	0.17	4.73×10^{-2}	None	Nutrient reservoir activity	None	11-S seed storage protein, plant
A0A3B6MQA1	RRM domain-containing protein OS=*Triticum aestivum*	6.96	22.54	4	1.20	0.05	4.00×10^{-2}	None	RNA binding	Ribonucleoprotein complex	Eukaryotic RNA-binding
A0A3B6EP73	Uncharacterized protein OS=*Triticum aestivum*	5.62	15.13	3	1.40	0.20	2.29×10^{-2}	None	None	None	None predicted
A0A3B6FKP0	PH domain-containing protein OS=*Triticum aestivum*	5.21	10.76	3	1.71	0.34	7.96×10^{-3}	None	None	None	Ricin B-like lectin EULS3-like
A0A3B6TCF4	Uncharacterized protein OS=*Triticum aestivum*	2.06	3.303	1	1.23	0.09	2.76×10^{-2}	SNARE complex assembly	SNARE binding	Endosome	None predicted
A0A3B5XZW3	Remorin C-terminal domain-containing protein OS=*Triticum aestivum*	2.02	6.78	1	-1.65	0.02	1.98×10^{-2}	None	None	None	None predicted

[a] Protein accession number from the UniProt database (http://www.uniprot.org).

[b] Protein name corresponding to the accession number from the UniProt database (http://www.uniprot.org).

[c] Protein score generated by ProteinPilot software relating to the confidence of protein identification.

[d] Percentage coverage is determined by the number of amino acids of sequenced peptides against the total length of the protein, with a threshold of at least 95% confidence.

[e] Sequenced peptide refers to number of peptides sequenced. All positively identified proteins were identified based on a single peptide.

[f] Ratio represents the average fold change. A negative value indicates down-regulation.

[g] Standard deviation obtained from comparing leaf protein control and treatment values.

[h] Probability-value of the quantified difference between the control and drought-stressed leaf proteins.

[i] Gene ontology analysis as predicted from the UniProt database (http://www.ebi.ac.uk/QuickGO/). P=Biological Process, F=Molecular Function, and C= Cellular Component.

j Family and superfamily names as predicted by InterPro database (http://www.ebi.ac.uk/interpro/).
