# Supplementary material for: Comparative Physiological, Biochemical, and Leaf Proteome Responses of Contrasting Wheat Varieties to Drought Stress
Source: Plants (Basel). 2024 Oct 5;13(19):2797. doi: 10.3390/plants13192797 (PMC11478804; doi:10.3390/plants13192797)
Supplement: Supplementary file 1 [file plants-13-02797-s001.zip › Tables S7.pdf]

Table S7: List of drought-responsive leaf proteins of the drought tolerant wheat variety BW35695 identified using iTRAQ and LC-MS/MS.

**Accession**[a]	**Protein name**[b]	**Scor**[c]	**% Cov**[d]	**Seq. Pep**[e]	**Ratio**[f]	**SD**[g]	**p-value**[h]	**Gene ontology analysis**[i]			**Family name**[j]
								**P**	**F**	**C**	
**Primary metabolism**											
A0A2X0S1W6	Glutamate synthase (ferrodoxin) OS=*Triticum aestivum*	28.93	11.77	18	-1.12	0.07	2.30×10^{-2}	Glutamate biosynthetic process	Glutamate synthase (NADH) activity	None	Electron transfer and nitrogen metabolism enzyme
A0A3B6PIU3	Enoyl reductase (ER) domain-containing protein OS=*Triticum aestivum*	26.37	46.74	15	1.34	0.12	6.13×10^{-3}	None	Oxidoreductase activity	None	NADPH-dependent oxidoreductase AOR-like
A0A3B6MJZ2	5-methyltetrahydropteroyltriglutamate--homocysteine S-methyltransferase OS=*Triticum aestivum*	22.4	18.05	12	1.37	0.18	7.84×10^{-3}	Methionine biosynthetic process	Methyltransferase activity	None	Cobalamin-independent methionine synthase
A0A3B6GU52	Glycine cleavage system P protein OS=*Triticum aestivum*	20.23	15.62	15	-1.17	0.06	1.35×10^{-2}	Glycine catabolic process	Glycine dehydrogenase (decarboxylating) activity	Mitochondrion	Glycine dehydrogenase (decarboxylating)
A0A3B6UC94	Acid phosphatase OS=*Triticum aestivum*	12.22	30.36	7	2.05	0.28	5.05×10^{-4}	None	Acid phosphatase activity	None	Acid phosphatase, plant
A0A3B6FKL0	Nucleoside phosphorylase domain-containing protein OS=*Triticum aestivum*	7.75	21.61	5	1.66	0.27	3.78×10^{-3}	Nucleoside metabolic process	Catalytic activity	None	Phosphorylase superfamily
A0A3B6H1E6	Glucan endo-1,3-beta-D-glucosidase OS=*Triticum aestivum*	7.2	22.26	4	1.21	0.17	4.73×10^{-2}	Carbohydrate metabolic process	Hydrolase activity, acting on glycosyl bonds	Plasma membrane	Glycoside hydrolase family 17, plant
A0A3B6MZI6	UDP-glucose 6-dehydrogenase OS=*Triticum aestivum*	4.76	11.46	4	1.25	0.18	3.78×10^{-2}	Glycosaminoglycan biosynthetic process	UDP-glucose 6-dehydrogenase activity	Nucleus	UDP-glucose 6-dehydrogenase, eukaryotic type
A0A1D5URN5	Fibronectin type III-LIKE domain-containing protein OS=*Triticum aestivum*	2.36	3.76	2	-1.15	0.05	3.59×10^{-2}	Xylan catabolic process	Xylan 1,4-beta-xylosidase activity	Extracellular region	Beta-D-xylosidase
A0A3B6B442	Aspartate/glutamate/uridylate kinase domain-containing protein OS=*Triticum aestivum*	2.07	3.86	1	-1.92	0.34	4.08×10^{-2}	Glutamine family amino acid biosynthetic process	Acetylglutamate kinase activity	Cytoplasm	Glutamate/ acetylglutamate kinase
A0A3B6TUD9	Thiamine thiazole synthase, chloroplastic OS=*Triticum aestivum*	2.05	5.98	2	-1.74	0.13	3.42×10^{-2}	Thiamine biosynthetic process	Pentosyltransferase activity	Chloroplast	Thiamine thiazole synthase

A0A3B6KK25	3-dehydroquinate synthase OS=*Triticum aestivum*	2.03	7.00	1	-1.42	0.07	1.38×10^{-2}	Amino acid family biosynthetic process	3-dehydroquinate synthase activity	Cytoplasm	Dehydroquinate synthase
A0A3B6NHD8	O-methyltransferase ZRP4 OS=*Triticum aestivum*	1.96	10.99	2	1.56	0.41	3.89×10^{-2}	Aromatic compound biosynthetic process	S-adenosylmethionine-dependent methyltransferase activity	None	O-methyltransferase COMT-type
A0A3B5ZXG4	Glycosyltransferase OS=*Triticum aestivum*	1.14	3.16	1	-1.50	0.12	2.23×10^{-2}	None	UDP-glycosyltransferase activity	None	UDP-glucuronosyl/ UDP-glucosyltransferas e
A0A3B6MS26	Glucose-6-phosphate 1-epimerase OS=*Triticum aestivum*	0.77	5.35	1	-2.37	0.15	1.86 × 10	Carbohydrate metabolic process	Glucose-6-phosphate 1-epimerase activity	Cytoplasm	Glucose-6-phosphate 1-epimerase
A0A3B6KPK9	Beta-glucosidase OS=*Triticum aestivum*	13.38	13.30	8	2.27	0.88	4.53×10^{-2}	Carbohydrate metabolic process	Beta-glucosidase activity	Extracellular region	Cellulase degradation glycosyl hydrolase 3
**Energy**											
P11383	Ribulose bisphosphate carboxylase large chain OS=*Triticum aestivum*	72.15	71.91	189	-1.24	0.10	3.23×10^{-2}	photosynthesis	Ribulose-bisphosphate carboxylase activity	Chloroplast	RuBisCO large subunit type I
P20858	ATP synthase subunit beta, chloroplastic OS=*Triticum aestivum*	55.08	67.87	82	-1.34	0.11	3.09×10^{-2}	ATP biosynthesis process	Proton-transporting ATP synthase activity, rotational mechanism	Chloroplast	ATPase alpha/beta chains
P12112	ATP synthase subunit alpha, chloroplastic OS=*Triticum aestivum*	54.35	49.01	62	-1.46	0.15	1.40×10^{-2}	ATP biosynthesis process	Proton-transporting ATP synthase activity, rotational mechanism	Chloroplast	ATPase alpha/beta chains
A0A3B6TID0	Transketolase OS=*Triticum aestivum*	35.39	39.19	30	-1.21	0.06	7.17×10^{-3}	Pentose-phosphate shunt	Transketolase activity	Cytosol	Transketolase, bacterial-like
P24065	Photosystem II CP47 reaction center protein OS=*Triticum aestivum*	31.02	30.71	39	-2.02	0.12	1.01×10^{-2}	Photosynthesis	Chlorophyll binding	Photosystem II	Photosystem II CP47 reaction centre protein
A0A3B6AYY2	23 kDa subunit of oxygen evolving system of photosystem II OS=*Triticum aestivum*	30.74	57.14	57	1.56	0.35	4.27×10^{-2}	Photosynthesis	Calcium ion binding	Photosystem II	PsbP
A0A3B5Z5T0	Malate dehydrogenase OS=*Triticum aestivum*	27.38	45.75	19	1.20	0.14	3.94 × 10	Malate metabolic process	L-malate dehydrogenase activity	Cytoplasm	Malate dehydrogenase, type 1

A0A3B6N1I7	Photosystem I reaction center subunit II, chloroplastic OS=*Triticum aestivum*	21.89	57.35	18	-1.57	0.19	1.60×10^{-2}	Photosynthesis	None	Photosystem I	Photosystem I PsaD
A0A3B6LQN1	Chlorophyll a-b binding protein, chloroplastic OS=*Triticum aestivum*	19.89	50.94	27	-1.63	0.11	2.36×10^{-2}	Photosynthesis	Chlorophyll binding	Chloroplast	Chlorophyll A-B binding protein
A0A3B6JMT4	Glyceraldehyde-3-phosphate dehydrogenase OS=*Triticum aestivum*	27.81	35.36	27	-2.20	0.42	4.92×10^{-2}	Glucose metabolic process	Glyceraldehyde-3-phosphate dehydrogenase activity	None	Glyceraldehyde-3-phosphate dehydrogenase, type 1
A0A3B6MII9	Phosphopyruvate hydratase OS=*Triticum aestivum*	18.38	30.94	9	1.19	0.07	4.48×10^{-3}	Glycolytic process	Phosphopyruvate hydratase activity	Phosphopyruvate hydratase complex	Enolase
W5GFA4	Chlorophyll a-b binding protein, chloroplastic OS=*Triticum aestivum*	18.15	48.33	49	-1.36	0.17	2.82×10^{-2}	Photosynthesis	Chlorophyll binding	Chloroplast	Chlorophyll A-B binding protein
W5C4P1	Uncharacterized protein OS=*Triticum aestivum*	14.58	33.18	14	-1.92	0.08	1.06×10^{-2}	Photosynthesis	Electron transporter, transferring electron within the cyclic electron transport pathway of photosynthesis activity	Chloroplast	Oxygen-evolving enhancer protein 3, plants
W5AY52	Chlorophyll a-b binding protein, chloroplastic OS=*Triticum aestivum*	14.2	40.78	10	-1.67	0.08	1.53×10^{-3}	Photosynthesis	Chlorophyll binding	Chloroplast	Chlorophyll A-B binding protein
W5D4R0	Photosystem II 22 kDa protein, chloroplastic OS=*Triticum aestivum*	13.37	28.31	7	-1.63	0.13	5.52×10^{-3}	None	None	Chloroplast thylakoid membrane	Chlorophyll A-B binding protein
A0A3B6MXE7	Photosystem I reaction center subunit III OS=*Triticum aestivum*	11.64	27.71	9	-1.36	0.09	5.36×10^{-3}	Photosynthesis	None	Photosystem I	Photosystem I PsaF, reaction centre subunit III
A0A3B6QKY1	Aconitate hydratase OS=*Triticum aestivum*	11.24	6.67	5	2.05	0.47	5.72×10^{-3}	Citrate metabolic process	Aconitate hydratase activity	Cytosol	Aconitase/Iron-responsive element-binding protein 2
P58386	Photosystem I P700 chlorophyll a apoprotein A2 OS=*Triticum aestivum* GN=psaB	10.27	11.85	11	-2.39	0.09	2.93×10^{-3}	Photosynthesis	Chlorophyll binding	Photosystem I	Photosystem I PsaB
A0A3B6QDB2	Photosystem II protein D1 OS=*Triticum aestivum*	10	20.70	7	-1.57	0.14	2.89×10^{-2}	Photosynthesis	Chlorophyll binding	Photosystem II	Photosynthetic reaction centre, L/M

P58311	Photosystem I P700 chlorophyll a apoprotein A1 OS=*Triticum aestivum* GN=psaA	10.09	6.53	7	-2.39	0.04	9.95×10^{-3}	Photosynthesis	Chlorophyll binding	Photosystem I	Photosystem I PsaA
A0A3B6HUR4	Chlorophyll a-b binding protein, chloroplastic OS=*Triticum aestivum*	9.39	20.14	7	-1.91	0.09	4.14×10^{-3}	Photosynthesis	Chlorophyll binding	Chloroplast	Chlorophyll A-B binding protein
A0A3B6AWZ1	Chlorophyll a-b binding protein, chloroplastic OS=*Triticum aestivum*	8.48	27.99	13	-1.58	0.05	3.46×10^{-3}	Photosynthesis	Chlorophyll binding	Chloroplast	Chlorophyll A-B binding protein
W5F8Z5	Chlorophyll a-b binding protein, chloroplastic OS=*Triticum aestivum*	8.93	34.22	10	-1.69	0.07	1.88×10^{-4}	Photosynthesis	Chlorophyll binding	Chloroplast	Chlorophyll A-B binding protein
A0A1D5V1G5	Chlorophyll a-b binding protein, chloroplastic OS=*Triticum aestivum*	7.56	21.68	7	-1.39	0.14	3.31×10^{-2}	Photosynthesis	Chlorophyll binding	Chloroplast	Chlorophyll A-B binding protein
A0A3B5Z4J5	ATP synthase subunit b, chloroplastic OS=*Triticum aestivum*	5.52	16.57	3	-1.55	0.09	2.09×10^{-2}	Proton motive force-driven ATP synthesis	Proton transmembrane transporter activity	Membrane	ATPase, FO complex, subunit b/b'
P69415	Photosystem I iron-sulfur center OS=*Triticum aestivum*	5.01	27.16	4	-1.65	0.06	3.97×10^{-2}	Photosynthesis	4 iron, 4 sulfur cluster binding	Photosystem I	Photosystem I protein PsaC
A0A3B6PUD8	Photosystem II 10 kDa polypeptide, chloroplastic OS=*Triticum aestivum*	4.99	27.14	4	-1.64	0.08	5.55×10^{-3}	Photosynthesis	None	photosystem II	Photosystem II PsbR
A0A3B6RH69	Chlorophyll a-b binding protein, chloroplastic OS=*Triticum aestivum*	4.47	17.41	6	-1.44	0.08	2.20×10^{-2}	Photosynthesis	Chlorophyll binding	Chloroplast	Chlorophyll A-B binding protein
A0A3B5ZZE4	Chlorophyll a-b binding protein, chloroplastic OS=*Triticum aestivum*	13.34	52.09	24	-1.44	0.12	2.91×10^{-2}	Photosynthesis	Chlorophyll binding	Chloroplast	Chlorophyll A-B binding protein
F6K744	Chlorophyll a-b binding protein, chloroplastic OS=*Triticum aestivum*	17.15	52.26	29	-2.27	0.06	1.71×10^{-3}	Photosynthesis	Chlorophyll binding	Chloroplast	Chlorophyll A-B binding protein
A0A3B6U9Q7	Cytochrome b/b6 C-terminal region profile domain-containing protein OS=*Triticum aestivum*	1.11	5.75	1	-3.44	0.21	1.90×10^{-2}	Photosynthesis	Ubiquinol-cytochrome-c reductase activity	Thylakoid	Cytochrome b6/f complex, subunit IV
**Protein synthesis/folding/degradation**											
A0A3B6TR29	ATP-dependent zinc metalloprotease OS=*Triticum aestivum*	25.44	24.52	14	-1.38	0.09	1.98×10^{-2}	Proteolysis	ATP-dependent peptidase activity	Membrane	ATP-dependent zinc metalloprotease, FtsH
W5ECL2	Large ribosomal subunit uL2 C-terminal domain-containing protein OS=*Triticum aestivum*	16.68	26.05	11	1.27	0.18	3.15×10^{-2}	Translation	Structural constituent of ribosome	Ribosome	Large ribosomal subunit protein uL2

A0A3B6QDU5	50S ribosomal protein L3, chloroplastic OS=*Triticum aestivum*	13.1	41.67	15	1.30	0.07	1.33×10^{-2}	Translation	Structural constituent of ribosome	Ribosome	Large ribosomal subunit protein uL3
A0A3B6JLK1	Large ribosomal subunit protein L15/eL18 domain-containing protein OS=*Triticum aestivum*	10.99	16.60	7	1.33	0.21	3.20×10^{-2}	Translation	Structural constituent of ribosome	Ribosome	Large ribosomal subunit protein uL15
W5D067	Small ribosomal subunit protein uS7c OS=*Triticum aestivum*	9.14	16.00	7	-1.41	0.06	3.16×10	Translation	Structural constituent of ribosome	Ribosome	Ribosomal protein uS7
W5ASA4	Uncharacterized protein OS=*Triticum aestivum*	8	37.66	6	1.61	0.41	4.51×10^{-2}	Translation	Structural constituent of ribosome	Ribosome	Small ribosomal subunit protein uS19
A0A3B6JPZ3	Chaperonin CPN60-2, mitochondrial OS=*Triticum aestivum*	8.04	7.45	4	-1.30	0.14	2.46×10^{-2}	Protein refolding	ATP-dependent protein folding chaperone	Mitochondrion	Chaperonin Cpn60/GroEL
W5GTW2	Large ribosomal subunit protein eL1 domain-containing protein OS=*Triticum aestivum*	6.4	21.64	3	1.38	0.23	2.92×10^{-2}	Translation	Structural constituent of ribosome	Ribosome	Large ribosomal subunit protein eL14
P11534	Large ribosomal subunit protein uL2cz/uL2cy OS=*Triticum aestivum*	6.33	16.12	3	1.25	0.10	4.02×10^{-2}	Translation	Structural constituent of ribosome	Ribosome	Large ribosomal subunit protein uL2
A0A3B6JIR3	Heat shock cognate 70kDa protein OS=*Triticum aestivum*	16.71	19.82	12	1.55	0.34	4.59×10^{-2}	Chaperone cofactor-dependent protein refolding	Protein folding chaperone	Cytoplasm	Heat shock protein 70 family
A0A3B6TJK6	Small ribosomal subunit protein uS10 domain-containing protein OS=*Triticum aestivum*	5.94	19.35	4	1.44	0.20	2.99×10^{-2}	Translation	Structural constituent of ribosome	Ribosome	Small ribosomal subunit protein uS10
A0A1D6DK86	40S ribosomal protein S24 OS=*Triticum aestivum*	5.77	24.82	3	1.34	0.20	3.03×10^{-2}	Translation	Structural constituent of ribosome	Ribosome	Small ribosomal subunit protein eS24
A0A3B6B204	Large ribosomal subunit protein uL23 N-terminal domain-containing protein OS=*Triticum aestivum*	5.46	15.91	4	1.59	0.16	5.55×10^{-4}	Translation	Structural constituent of ribosome	Ribosome	Large ribosomal subunit protein uL23
W5D739	KOW domain-containing protein OS=*Triticum aestivum*	5.29	17.20	4	1.64	0.24	4.49×10^{-3}	Translation	Structural constituent of ribosome	Ribosome	Large ribosomal subunit protein uL24
Q93XQ6	Peptidyl-prolyl cis-trans isomerase OS=*Triticum aestivum*	5.37	26.32	9	1.41	0.11	1.21×10^{-2}	Protein folding	Peptidyl-prolyl cis-trans isomerase activity	Cytoplasm	Cyclophilin-type peptidyl-prolyl cis-trans isomerase

A0A3B6MTE3	Peptidylprolyl isomerase OS=*Triticum aestivum*	3.87	14.41	2	1.64	0.38	3.20×10^{-2}	None	Peptidyl-prolyl cis-trans isomerase activity	None	Peptidyl-prolyl cis-trans isomerase FKBP18-like
W5D7B8	60S ribosomal protein L18a OS=*Triticum aestivum*	3.75	12.92	2	1.40	0.20	3.92×10^{-2}	Translation	Structural constituent of ribosome	Ribosome	Large ribosomal subunit protein eL20
A0A3B6HYY4	Dipeptiylpeptidase IV N-terminal domain-containing protein OS=*Triticum aestivum*	3.75	5.16	3	1.37	0.14	3.39×10^{-3}	None	Serine-type peptidase activity	None	None predicted
A0A3B6N0K3	50S ribosomal protein L17, chloroplastic OS=*Triticum aestivum*	3.17	9.42	2	1.51	0.21	3.22×10^{-3}	Translation	Structural constituent of ribosome	Ribosome	Large ribosomal subunit protein bL17
A0A3B6N3E4	Sigma 54 modulation/S30EA ribosomal protein C-terminal domain-containing protein OS=*Triticum aestivum*	1.91	6.64	2	1.33	0.14	1.06×10^{-2}	Negative regulation of translation elongation	Ribosomal small subunit binding	Cytosolic small ribosomal subunit	Ribosome hibernation promoting factor, long/plastid
A0A3B6A2B9	60S ribosomal protein L37a, OS=*Triticum aestivum*	2	16.84	2	2.54	0.42	9.27×10^{-3}	Translation	Structural constituent of ribosome	Ribosome	Large ribosomal subunit protein eL43
A0A3B6KFH9	Uncharacterized protein OS=*Triticum aestivum*	6.42	17.06	4	1.26	0.06	4.33×10^{-2}	Translation	Structural constituent of ribosome	Ribosome	Large ribosomal subunit protein uL22
W5FV77	40S ribosomal protein S23 OS=*Triticum aestivum*	1.41	7.75	1	1.21	0.15	4.87×10^{-2}	Translation	Structural constituent of ribosome	Ribosome	Small ribosomal subunit protein uS12
A0A341U9I2	Small ribosomal subunit protein uS2c OS=*Triticum aestivum*	1.28	5.34	1	-1.40	0.10	2.10×10^{-2}	Translation	Structural constituent of ribosome	Ribosome	Small ribosomal subunit protein uS2
A0A3B6QGX5	60S ribosomal protein L6 OS=*Triticum aestivum*	2.22	10.08	2	1.65	0.29	4.00×10^{-2}	Translation	Structural constituent of ribosome	Ribosome	Large ribosomal subunit protein eL6
A0A3B6UD00	50S ribosomal protein L20 OS=*Triticum aestivum*	0.77	5.88	1	1.83	0.45	1.28×10^{-2}	Translation	Structural constituent of ribosome	Ribosome	Large ribosomal subunit protein bL20
W5D1D3	30S ribosomal protein S20, chloroplastic OS=*Triticum aestivum*	1.1	3.19	1	1.36	0.22	4.40×10^{-2}	Translation	Structural constituent of ribosome	Ribosome	Small ribosomal subunit protein bS20
**Transporters**											
A0A3B6LLS3	Uncharacterized protein OS=*Triticum aestivum*	7.96	19.64	5	1.29	0.18	4.38×10^{-2}	Monoatomic anion transmembrane transport	Voltage-gated monoatomic anion channel activity	Mitochondrion	Porin, eukaryotic type

A0A3B6GKQ2	Non-specific lipid-transfer protein OS=*Triticum aestivum*	4.17	10.67	2	1.71	0.40	1.31×10^{-2}	Lipid transport	Lipid binding	None	Plant non-specific lipid-transfer protein/Par allergen
A0A3B5YX09	Chloroplast inner envelope protein OS=*Triticum aestivum*	5.86	12.11	4	-1.50	0.10	2.53×10^{-2}	Protein import into chloroplast stroma	None	TOC-TIC super complex I	Protein TIC110, chloroplastic
A0A3B6I0D3	STI1/HOP DP domain-containing protein OS=*Triticum aestivum*	2.66	3.11	1	1.74	0.39	1.84×10^{-2}	Protein targeting to chloroplast	Chloroplast targeting sequence binding	None	None predicted
**Transcription**											
A0A3B6NX48	Histone H2A OS=*Triticum aestivum*	6.62	35.00	6	1.45	0.13	1.51×10^{-2}	Heterochromatin formation	Nucleosomal DNA binding	Nucleus	Histone H2A
A0A3B6NJ44	STI1 domain-containing protein OS=*Triticum aestivum*	4.49	5.59	2	1.47	0.34	4.15×10^{-2}	Chaperone cofactor-dependent protein refolding	Hsp70 protein binding	Histone deacetylase complex	None predicted
A0A3B6LSN3	MBD domain-containing protein OS=*Triticum aestivum*	3.5	5.68	2	1.52	0.18	7.18×10^{-3}	Transcription regulation	DNA binding	Nucleus	Methyl-CpG-binding domain-containing protein 10/11
Q8LRU5	HMG-I/Y protein HMGa OS=*Triticum aestivum*	2.94	5.29	1	1.60	0.40	3.88×10^{-2}	Negative regulation of DNA recombination	Nucleosomal DNA binding	Nucleus	High mobility group protein HMGA
Q43312	Protein H2A.7 OS=*Triticum aestivum*	4.27	12.69	2	-1.37	0.13	2.44×10^{-2}	Heterochromatic formation	Nucleosomal DNA binding	Nucleus	Histone H2A
A0A3B6MXZ6	H15 domain-containing protein OS=*Triticum aestivum*	2	7.45	1	1.84	0.44	4.79×10^{-2}	Negative regulation of DNA recombination	Nucleosomal DNA binding	Nucleus	Linker histone H1/H5
A0A3B6GNG0	Histone H2B OS=*Triticum aestivum*	29.6	69.12	54	1.53	0.24	5.14×10^{-3}	None	DNA binding	Nucleus	Histone H2B
A0A3B6QBC0	Histone H2B OS=*Triticum aestivum*	31.04	69.66	51	1.34	0.19	2.53×10^{-2}	None	DNA binding	Nucleus	Histone H2B
**Defence/ROS detoxification**											
A0A3B6C505	(S)-2-hydroxy-acid oxidase OS=*Triticum aestivum*	21.9	38.30	14	-1.49	0.09	1.15×10^{-2}	Response to other organism	(S)-2-hydroxy-acid oxidase activity	Peroxisome	Alpha-hydroxy acid dehydrogenase, FMN-dependent
F1DKC1	Catalase OS=*Triticum aestivum*	15.61	18.42	7	1.48	0.20	1.39×10^{-2}	Response to oxidative stress	Catalase activity	Cytoplasm	Catalase
A0A3B6PS76	Thioredoxin domain-containing protein OS=*Triticum aestivum*	6.72	10.26	5	1.22	0.08	1.69×10^{-2}	None	Oxidoreductase activity	None	Thioredoxin-like protein CDSP32

A0A3B6EFA0	Uncharacterized protein OS=*Triticum aestivum*	6.58	17.31	3	2.66	0.73	4.24×10	Regulation of abscisic acid biosynthetic process	None	None	Nodulin-related protein ½ family
A0A3B6HWK9	Germin-like protein OS=*Triticum aestivum*	5.9	10.05	3	1.44	0.29	4.12×10^{-2}	None	Manganese ion binding	Apoplast	Germin
Q8W428	Chitinase OS=*Triticum aestivum*	4.16	18.27	3	-1.56	0.17	9.40×10^{-3}	Chitin catabolic process	Chitinase activity	None	Glycoside hydrolase, family 19
A0A3B6C7J3	Ultraviolet-B receptor UVR8 OS=*Triticum aestivum*	2.13	3.17	1	1.22	0.09	1.59×10^{-2}	None	None	None	Ubiquitin ligases and GTPase regulators
A0A3B6C860	Peroxidase OS=*Triticum aestivum*	2.04	2.48	1	1.39	0.14	5.84×10^{-3}	Response to oxidative stress	Peroxidase activity	Extracellular region	Plant peroxidase
A0A172WCB1	Cold-responsive LEA/RAB-related COR protein OS=*Triticum aestivum*	6.95	29.52	6	3.23	1.19	1.01×10^{-2}	None	None	None	None predicted
A0A3B6TZ07	GH18 domain-containing protein OS=*Triticum aestivum*	1.96	2.95	1	1.84	0.28	1.98×10^{-3}	Carbohydrate metabolic process	Chitinase activity	Extracellular region	Glycoside hydrolase 18 family chitinases
A0A3B6MJX1	Pathogen-related protein OS=*Triticum aestivum*	1.36	6.38	1	-2.00	0.13	1.68×10^{-2}	None	None	None	Pathogen-related defense protein
**Secondary metabolism**											
A0A3B6TV37	Amine oxidase domain-containing protein OS=*Triticum aestivum*	8.61	10.54	5	1.67	0.33	2.10×10^{-2}	Spermine catabolic process	Oxidoreductase activity	None	Flavin monoamine oxidase and related enzymes
A0A3B6QDX1	Delta-aminolevulinic acid dehydratase OS=*Triticum aestivum*	2.29	6.61	1	-1.59	0.17	8.35×10^{-3}	Chlorophyll biosynthetic process	Porphobilinogen synthase activity	Cytosol	Delta-aminolevulinic acid dehydratase family
A0A3B5Y2F9	Dienelactone hydrolase domain-containing protein OS=*Triticum aestivum*	4.37	11.25	2	1.60	0.22	2.30×10^{-3}	None	Hydrolase activity	None	Dienelactone hydrolase family
**Cell structure**											
W5FAY5	Actin OS=*Triticum aestivum*	11.81	22.55	8	-1.50	0.15	3.53×10^{-2}	None	ATP binding	Cytoskeleton	Actin family
**Unclear classification**											
A0A3B6LG11	NAD-dependent epimerase/dehydratase domain-containing protein OS=*Triticum aestivum*	16.89	23.08	10	-1.23	0.08	4.60×10^{-3}	rRNA processing	RNA binding	Cytosol	None predicted
A0A3B6MQA1	RRM domain-containing protein OS=*Triticum aestivum*	10.67	23.36	8	-1.24	0.12	4.05×10^{-2}	None	RNA binding	Ribonucleoprotein complex	Eukaryotic RNA-binding

A0A3B6LS14	Cupin type-1 domain-containing protein OS=*Triticum aestivum*	5.89	12.57	4	1.38	0.18	3.28×10^{-2}	None	None	None	Seed storage and functional proteins
A0A3B6GTL4	DJ-1/PfpI domain-containing protein OS=*Triticum aestivum*	3.84	7.98	2	-1.79	0.11	5.38×10^{-4}	Glyoxal metabolic process	None	Cytoplasm	Protein/nucleic acid deglycase DJ-1
A0A3B6TRL4	Thylakoid membrane protein slr0575 OS=*Triticum aestivum*	3.34	8.40	2	-1.78	0.19	1.58×10^{-2}	None	None	Membrane	Protein of unknown function DUF2854
A0A3B6AVR1	Uncharacterized protein OS=*Triticum aestivum*	2	7,34	1	-1.63	0,06	4.05×10^{-2}	None	Deaminase activity	Cytosol	RidA family
A0A3B6N353	Pentacotripeptide-repeat region of PRORP domain-containing protein OS=*Triticum aestivum*	1.32	1.47	1	2.10	0.41	3.42×10^{-3}	None	None	None	Tetratricopeptide-like helical domain superfamily
W5CRR3	DUF538 domain-containing protein OS=*Triticum aestivum*	1.03	12.43	2	-2.38	0.13	1.91×10^{-3}	None	None	None	Protein of unknown function DUF538
A0A3B5ZXF0	Protein kinase domain-containing protein OS=*Triticum aestivum*	0.83	1.84	1	1.92	0.44	9.42×10^{-3}	None	Protein kinase activity	None	None predicted

[a] Protein accession number from the UniProt database (http://www.uniprot.org).

[b] Protein name corresponding to the accession number from the UniProt database (http://www.uniprot.org).

[c] Protein score generated by ProteinPilot software relating to the confidence of protein identification.

[d] Percentage coverage is determined by the number of amino acids of sequenced peptides against the total length of the protein, with a threshold of at least 95% confidence.

[e] Sequenced peptide refers to number of peptides sequenced. All positively identified proteins were identified based on a single peptide.

[f] Ratio represents the average fold change. A negative value indicates down-regulation.

[g] Standard deviation obtained from comparing leaf protein control and treatment values.

[h] Probability-value of the quantified difference between the control and drought-stressed leaf proteins.

[i] Gene ontology analysis as predicted from the UniProt database (http://www.ebi.ac.uk/QuickGO/). P= Biological Process, F= Molecular Function, and C=Cellular Component.

[j] Family and superfamily names as predicted by InterPro database (http://www.ebi.ac.uk/interpro/).
