# Supplementary figures and images for: Comparative Physiological, Biochemical, and Leaf Proteome Responses of Contrasting Wheat Varieties to Drought Stress
Source: Plants (Basel). 2024 Oct 5;13(19):2797. doi: 10.3390/plants13192797 (PMC11478804; doi:10.3390/plants13192797)

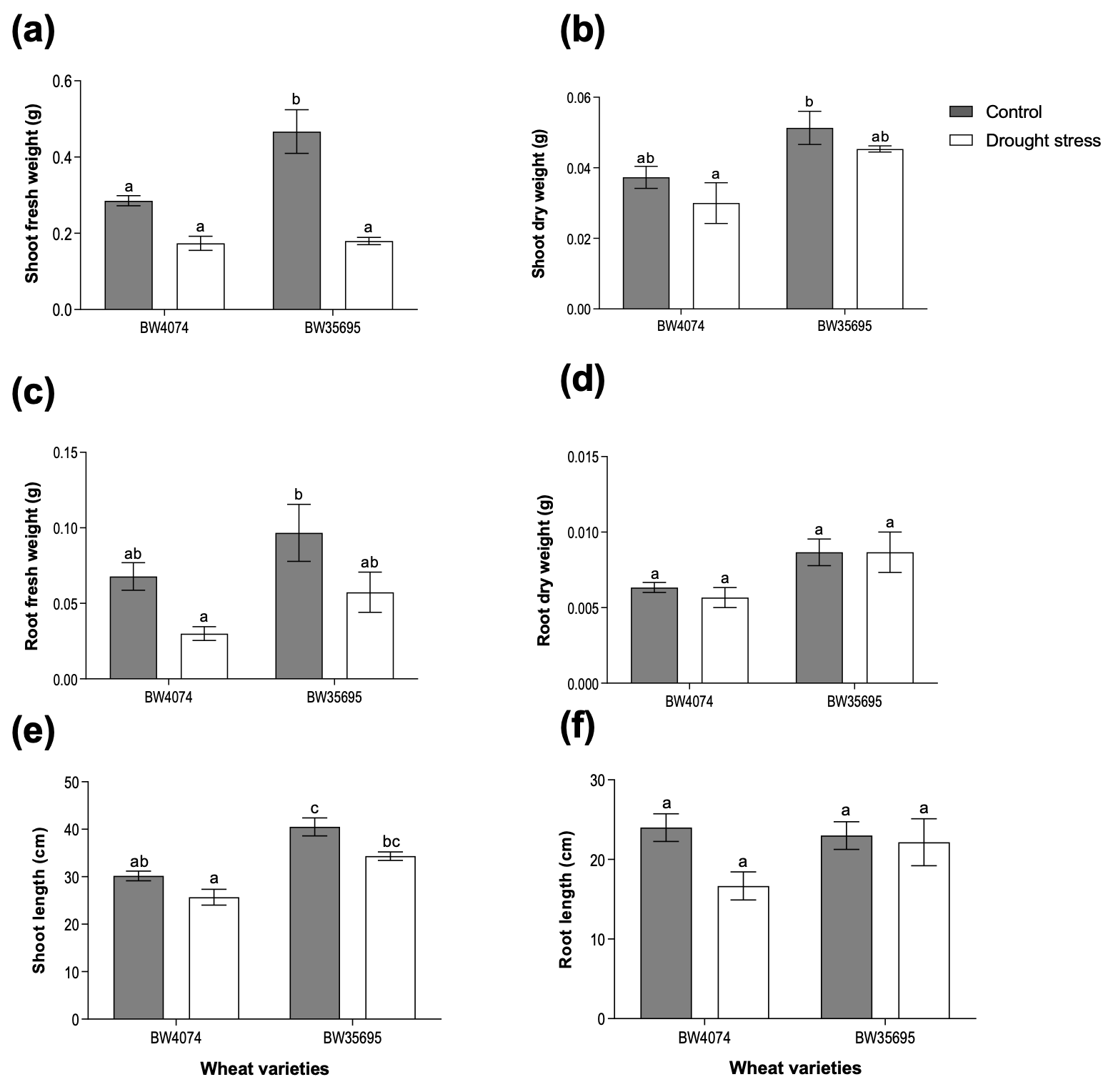

Supplement: Supplementary file 1 [file plants-13-02797-s001.zip › Figure S1.png]

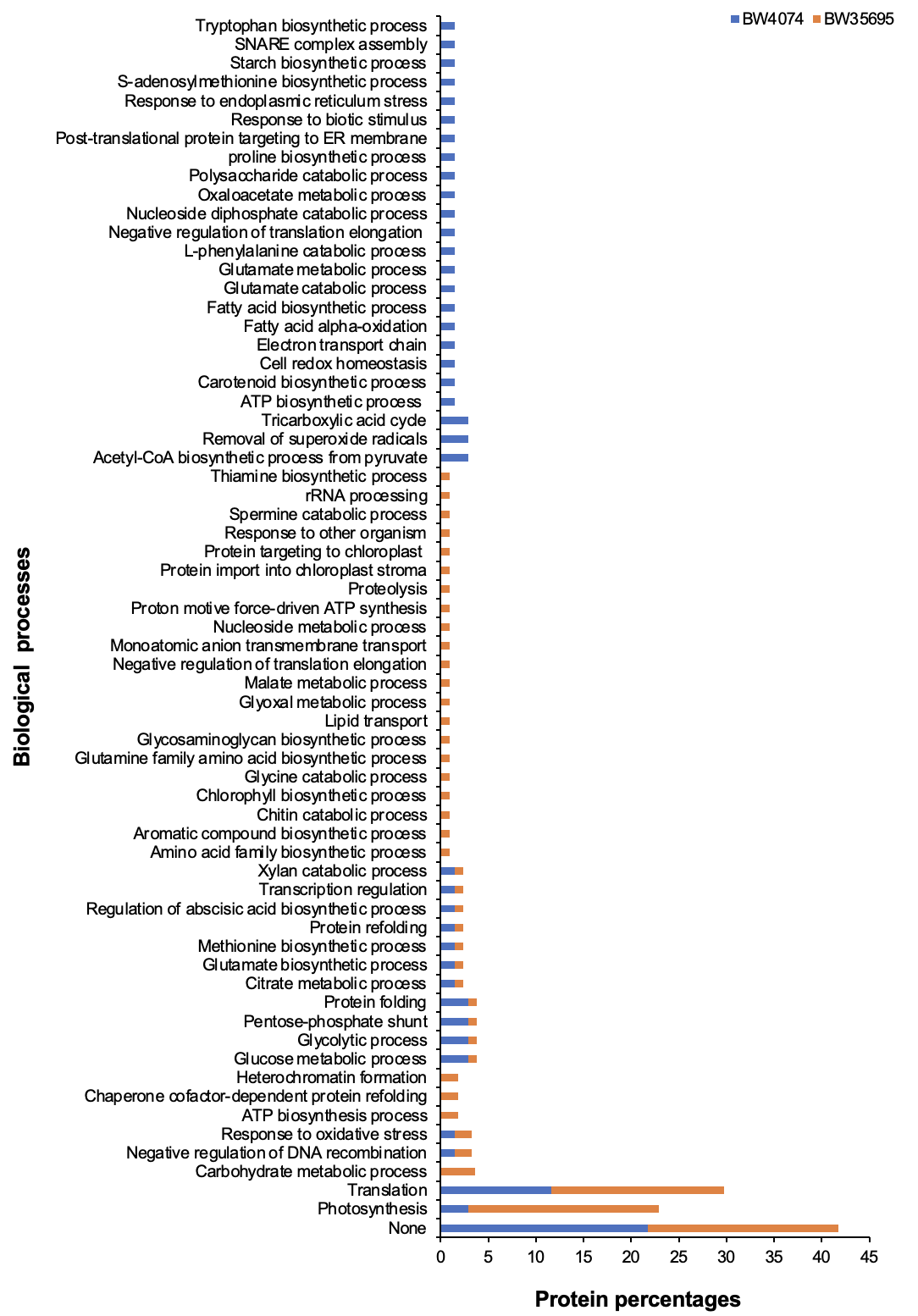

Supplement: Supplementary file 1 [file plants-13-02797-s001.zip › Figure S2.png]

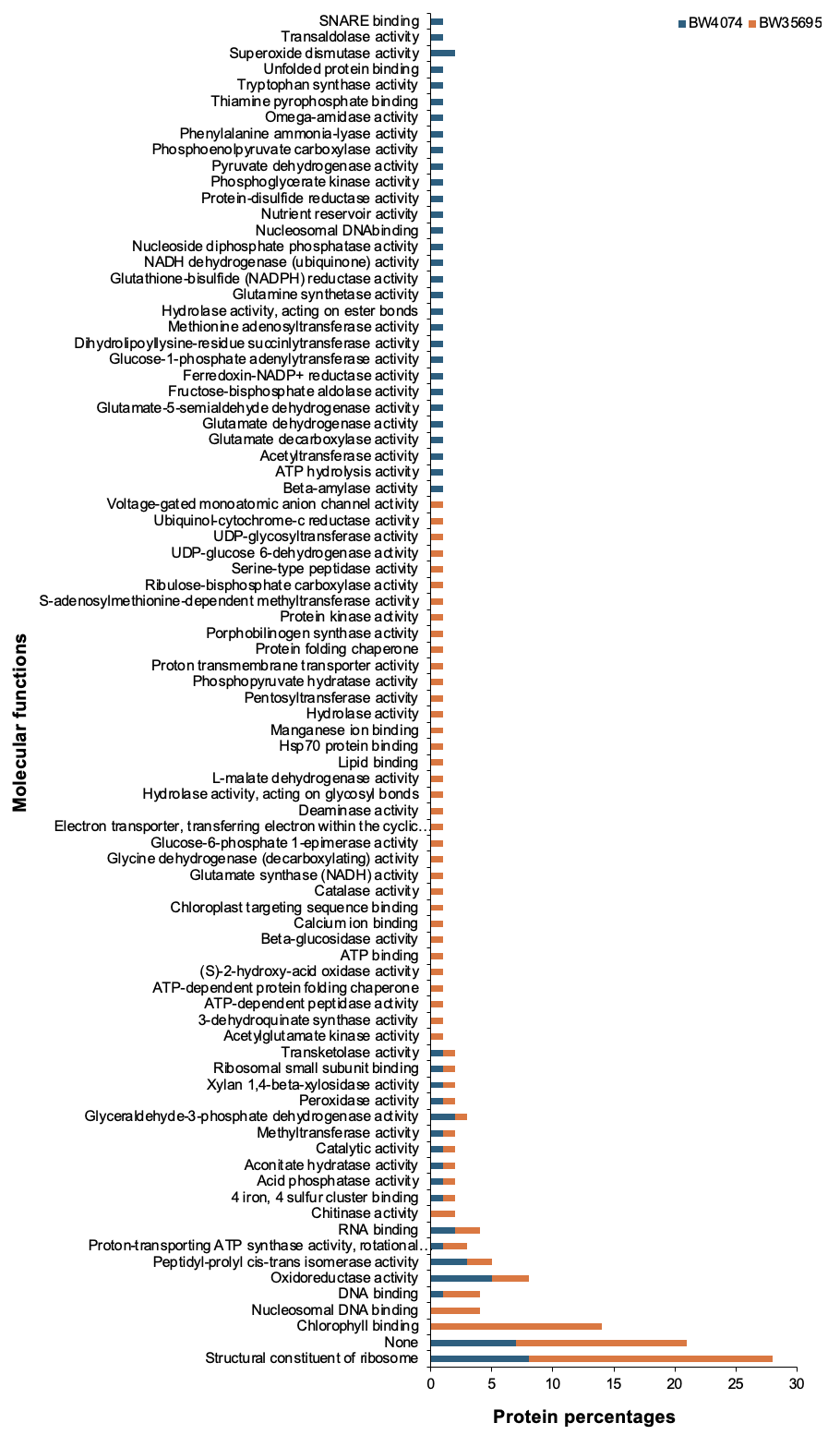

Supplement: Supplementary file 1 [file plants-13-02797-s001.zip › Figure S3.png]
